# UHPLC-QTOF-MS/MS profiling, molecular networking, and molecular docking analysis of *Gliricidia sepium* (Jacq.) Kunth. ex. Walp. stem ethanolic extract and its gastroprotective effect on gastritis in rats

**DOI:** 10.1016/j.toxrep.2025.101944

**Published:** 2025-02-04

**Authors:** Aya A. Wafaey, Seham S. El-Hawary, Osama G. Mohamed, Sahar S. Abdelrahman, Alaa M. Ali, Ahmed A. El-Rashedy, Mohamed F. Abdelhameed, Farid N. Kirollos

**Affiliations:** a Pharmacognosy Department, Faculty of Pharmacy, Cairo University, Kasr El-Aini, Cairo 11562, Egypt; b Natural Products Discovery Core, Life Sciences Institute, University of Michigan, Ann Arbor, MI 48109, USA; c Department of Pathology, Faculty of Veterinary Medicine, Cairo University, Giza 12211, Egypt; d Natural and Microbial Products Department, National Research Center, 33 El-Bohouth St., Dokki, Cairo 12622, Egypt; e Pharmacology Department, Medical Research and Clinical Studies Institute, National Research Centre, 33 El-Bohouth St., Dokki, Cairo 12622, Egypt

**Keywords:** *Gliricidia sepium* (Jacq.) Kunth. ex. Walp., Plant stem, UPLC-MS, Cluster analysis, Molecular docking simulation, Gastroprotective agents, I-kappa B proteins

## Abstract

Metabolic profiling of the crude ethanolic extract of *Gliricidia sepium* (Jacq.) Kunth. ex. Walp. stem ethanolic extract (GSS) was conducted using ultra-high performance quadrupole time of flight mass spectrometry/mass spectrometry (UHPLC-QTOF-MS/MS) in negative mode, resulting in the identification of 23 compounds belonging to various classes such as flavonoids, fatty acids, triterpenoid saponins, and phenolic acids. Notably, eight flavonoids including kaempferol-3-*O*-robinoside-7-*O*-rhamnoside, isoquercitrin, kaempferol-3-*O*-rutinoside, apigenin-7-glucoside, kaempeferol-7-*O*-rhamnoside, luteolin, apigenin, and liquiritigenin, along with two phenolic acids (4-hydroxycinnamic acid and 2-hydroxyhydrocinnamic acid) and four triterpenoid saponins (soyasaponin I, soyasaponin II, soyasaponin III, and kaikasaponin III) were dereplicated. Additionally, nine fatty acid derivatives were identified, including azelaic acid and 2-isopropyl malic acid. Molecular networking analysis revealed the formation of clusters among compounds while others do not form clusters. Further analysis indicated that the GSS ethanolic extract exhibited a total phenolic content of 38.78 ± 1.609 µg of gallic acid equivalent/mg and a total flavonoid content of 5.62 ± 0.50 µg of rutin equivalent/mg. Biological evaluations showed that GSS ethanolic extract mitigated gastric tissue injury induced by pyloric ligation, with a notable reduction in oxidative stress marker reactive oxygen species levels and inflammatory cytokines interleukin-6 and tumor necrosis factor-alpha levels. Additionally, it enhanced superoxide dismutase and inhibitor of nuclear factor kappa B alpha levels, while lowering the expression of inducible nitric oxide synthase. Histopathological examination revealed significant improvements in gastric tissue morphology in GSS-treated groups compared to the control group. Molecular docking studies indicated potential interactions between GSS ethanolic extract compounds and various target proteins involved in oxidative stress, inflammation, and gastric protection in gastritis. This study aims to investigate the potential gastroprotective activity of GSS ethanolic extract against gastritis induced via pyloric ligation.

## Highlights

• Our conclusion: The current study highlights the tight relationship between the presence of flavonoids, phenolic acids, and saponins such as kaempferol-3-*O*-rutinoside-7-*O*-rhamnoside, soyasaponin III, and soyasaponin I which are identified as the major compounds in *Gliricidia sepium* (Jacq.) Kunth. ex. Walp. stem ethanolic extract through UHPLC-QTOF-MS/MS analysis and possess antioxidant and anti-inflammatory properties suggested for diabetic wound healing activity.
• The incorporation of antioxidant and anti-inflammatory parameters and pathways via IL-6, ROS, SOD, iNOS, IκB*α*, and TNF-*α*.
• Molecular Networking for compounds identified by UHPLC-QTOF-MS/MS.
• Molecular Docking for various target proteins such as IL-6, IL-10, HO-1, SOD, and keap-1.
• The promising gastroprotective effect against gastritis.

## 1 Introduction

Gastritis is a medical condition distinguished by a state of inflammation, irritation, or erosion of the stomach lining which may manifest either suddenly as an acute gastritis or develop gradually as chronic gastritis. The diagnosis of gastritis primarily relies on identifying typical symptoms such as abdominal pain, indigestion, abdominal bloating, nausea, and vomiting [1]. The prevalence of gastritis is markedly lower in developed countries, with 34.7 % of the population in contrast to the higher rate of 50.8 % in developing countries [2].

The main causes of gastritis include alcohol consumption, prolonged use of non-steroidal anti-inflammatory drugs (NSAIDs) such as aspirin or ibuprofen, infection with *Helicobacter pylori*, chronic bile reflux, stress, autoimmune disorders, and smoking [3]. Its complications include gastric ulcer, gastric cancer, gastric bleeding, and anemia [4], [5]. Numerous plants have been utilized in the treatment of gastritis due to their phytochemical constituents, known for their antioxidant and anti-inflammatory properties [6]. Among these plants is *Gliricidia sepium* (Jacq.) Kunth. ex. Walp.

*Gliricidia sepium* (Jacq.) Kunth. ex. Walp. is a member of the Fabaceae family, which is renowned for its abundance of flavonoids and phenolic compounds [7]. This plant has exhibited noteworthy antioxidant and anti-inflammatory properties [8]. As the third-largest plant family after the Orchidaceae and Asteraceae, the Fabaceae family includes over 700 genera and approximately 20000 species [9] and is widely acknowledged for its significance in medicine [10]. The most reported activities associated with this family are antioxidant and anti-inflammatory activities [11]. Consequently, *Gliricidia sepium* (Jacq.) Kunth. ex. Walp. displays potential as a promising strategy for treating gastritis.

Various models have been developed to induce gastritis in rats to mimic human gastritis. Pyloric ligation is one of these models used to induce gastritis via a surgical procedure in rats. It involves the surgical occlusion of the pylorus, which is the junction between the stomach and the duodenum, leading to an accumulation of gastric secretions within the stomach. This accumulation of gastric juices can result in gastritis [12]. Pyloric ligation was first described by Shay et al., 1945 [13] which is a widely used method to induce gastritis and gastric ulcers in rats to study anti-ulcer drugs. This model is employed in preclinical research to evaluate anti-ulcer drugs (proton pump inhibitors and H_2_ blockers), natural compounds, or extracts with gastroprotective activity [14].

Many treatments have proven their effectiveness in decreasing gastritis occurrences, including proton pump inhibitors (such as omeprazole), prostaglandin analogs, and H_2_ receptor blockers (like cimetidine, ranitidine, and famotidine) [15]. There is a demand for the development of more affordable drugs with fewer side effects, especially from natural sources. Plants can produce better results because they focus on the causes of gastritis and manage the symptoms with their phytochemical constituents [16]. Currently, there is no information available regarding the antioxidant and anti-inflammatory effects of *Gliricidia sepium* (Jacq.) Kunth. ex. Walp. stem (GSS) ethanolic extract on gastritis. Hence, this study was conducted to evaluate GSS ethanolic extract potential for antioxidant and anti-inflammatory effects in gastritis.

## 2 Materials and methods

### 2.1 Plant material and collections

The stems of *Gliricidia sepium* (Jacq.) Kunth. ex. Walp. were procured from Mazhar Botanical Garden in July 2021. The identification of the plant was carried out by Therease Labib, a consultant of plant taxonomy at El-Orman Botanical Garden in Giza, Egypt. A particular sample of the stems was deposited in the herbarium of the Department of Pharmacognosy at Cairo University's Faculty of Pharmacy and designated with the voucher specimen number 15–8–2021. The study adhered to the occupational health and safety guidelines established by the scientific research and ethics committee of the Faculty of Pharmacy, Cairo University, in Giza, Egypt.

### 2.2 Preparation of the plant extract samples

Fresh stems of *Gliricidia sepium* (Jacq.) Kunth. ex. Walp. were soaked in 70 % ethanol to extract their constituents. The stems were combined with the solvent using a blender, and the ethanol was subsequently eliminated under vacuum conditions, ensuring that the temperature remained below 50 ℃. The dry extracts were preserved at −20°C for subsequent examination.

### 2.3 UHPLC-QTOF-MS/MS profiling of *Gliricidia sepium* (Jacq.) Kunth. ex. Walp. stem ethanolic extract

The utilization of the Agilent LC-MS system, which comprises an Agilent 1290 Infinity II UHPLC and an Agilent 6545 ESI-Q-TOF-MS in negative mode, was executed to procure ultra-high-performance liquid chromatography (UHPLC). The analysis of aliquots (1 µL) of ethanolic extracts (2 mg/mL in MeOH) was conducted through utilization of a Kinetex phenyl-hexyl (1.7 µm, 2.1 × 50 mm) column, which involved a 1 min isocratic elution of 90 % A (A: 100 % H_2_O + 0.1 % formic acid), followed by a 6 min linear gradient elution to 100 % B (95 % MeCN + 5 % H_2_O + 0.1 % formic acid) at a flow rate of 0.4 mL/min. The ESI conditions were set with a capillary temperature of 320°C, source voltage of 3.5 kV, and a sheath gas flow rate of 11 L/min. Ions possessing an intensity above 1000 counts at 6 scans/s, an isolation width of 1.3 ∼*m/z*, and a maximum of 9 selected precursors per cycle were detected in the full scan using ramped collision energy (5 × *m/z*/100 + 10 eV). In negative mode, the TFA C_2_HF_3_O_2_[M − H] ^−^ ion (*m/z* 112.985587) and hexakis (1 H,1 H,3H-tetrafluoropropoxy)-phosphazene C_18_H_18_F_24_N_3_O_6_P_3_ [M + TFA − H] ^−^ ion (*m/z* 1033.988109) were employed as internal lock masses [17].

### 2.4 Molecular networking

The molecular network (MN) was constructed using the UHPLC-QTOF-MS/MS data in the negative from *Gliricidia sepium* (Jacq.) Kunth. ex. Walp. stem ethanolic extract. The Global Natural Product Social Molecular Networking online (GNPS) (http://gnps.uscd.edu) is used to create MN. The MN parameters in GNPS to create clusters were as follows: minimum pairs cosine score at 0.70; fragment ion mass tolerance at 0.5 Da, precursor ion mass tolerance at 2.0 Da, minimum matched fragment ions at 6, and a minimum cluster size of 2. Cystoscope (ver. 3.10.1.) was used for network visualization and analysis and ChemDraw Ultra 11.0 was used to draw the structures.

### 2.5 Determination and preparation of samples and standards for total phenolic and flavonoid contents of *Gliricidia sepium* (Jacq.) Kunth. ex. Walp. stem ethanolic extract

The quantification of total phenolic compounds in ethanolic extract of GSS was conducted through the employment of the Folin-Ciocalteu colorimetric method, as explained by Attard in 2013 [18]. This involved the blending of 10 μL of either sample or standard with 100 μL of Folin-Ciocalteu reagent, which had previously undergone a 1:10 dilution. The concoction was subsequently introduced into a 96-well microplate, followed by the addition of 80 μL of 1 M Na_2_CO_3_. Incubation in the dark at room temperature of 25°C was affected for 20 minutes. Following this period, the resulting blue complex hue was evaluated at 630 nm, with the data expressed as means ± SD.

The quantification of total flavonoid in ethanolic extract of GSS was carried out by implementing the aluminum chloride method described by Kiranmai et al. in 2011 [19], with minor modifications to be executed in microplates. Specifically, 15 μL of either sample or standard was placed in a 96-well microplate, to which 175 μL of methanol and 30 μL of 1.25 % AlCl_3_ were added. Finally, 30 μL of 0.125 M C_2_H_3_NaO_2_ was added and incubated for 5 minutes. After the incubation period, the resulting yellow color was assessed at 420 nm, with the data expressed as means ± SD.

In the assessment of total phenolic content, the samples were formulated to achieve a concentration of 2 mg/mL. In contrast, for the evaluation of total flavonoid content, the samples were meticulously prepared at a concentration of 5 mg/mL using methanol as the solvent.

To determine the total phenolic content, a gallic acid stock solution of 1 mg/mL was meticulously prepared using methanol. Subsequently, a series of dilutions were generated, resulting in concentrations of 50, 100, 200, 400, 600, and 800 µg/mL.

For the assessment of total flavonoid content, a stock solution of standard Rutin was established, with a concentration of 200 µg/mL in methanol. From this stock solution, a set of dilutions was methodically prepared, yielding concentrations of 7.8125, 15.625, 31.25, 62.5, 125, 250, 500, and 1000 µg/mL.

### 2.6 Bioassay

#### 2.6.1 Drugs and chemicals

Omez® (Omeprazole) is a pharmaceutical product manufactured by Pharaonia Pharmaceuticals Industries in Borg AlArab, Alexandria, Egypt. Superoxide Dismutase (SOD, Catalog # K335–100) was obtained from Biovision Inc., Milpitas, CA, USA, Interleukin-6(IL-6) (Cloud-clone Corp., TX, USA, Catalog # SEA079Ra), Reactive Oxygen Species(ROS)(EIAab Inc., Wuhan, Hubei Province, China, Catalog # E1924r), and Tumor Necrosis Factor Alpha (TNF-*α*)(BioLegend Inc., San Diego, CA, USA, Catalog # 438204). Additionally, PCR was utilized to determine the expression of Inducible Nitric Oxide Synthase(iNOS)(Novus Biologicals LLC., Centennial, Colorado, USA, Catalog # NBP2–80257) and Inhibitor of Nuclear Factor Kappa B Alpha (IκB*α*) (Santa Cruz Biotechnology, Inc., Dallas, TX, USA, Catalog # SC-847). All other chemicals used in the experiment were of high analytical grade.

#### 2.6.2 Experimental animals

Sprague Dawley rats weighing between 150 and 200 g were sourced from the animal house colony situated at the National Research Centre in Dokki, Giza. To ensure stable conditions, the rats were acclimated in the laboratory room for at least one week. These conditions encompassed maintaining a room temperature within the range of 24–27ºC and adhering to a schedule of alternating 12-hour cycles of light and darkness. The rats were provided unrestricted access to a standard pellet diet and water, except when specific conditions required otherwise. All procedures involving the animals adhered to the guidelines set by the Institutional Ethics Committee and followed the recommended protocols for the proper care and utilization of laboratory animals.

#### 2.6.3 Induction of gastritis via pyloric ligation

Rats were kept in individual cages with raised mesh bottoms and deprived of food but were allowed free access to tap water for 18 hours before the experiments. Under thiopental anesthesia, the abdomen was incised along the middle, and then both the pylorus and junction between the forestomach and corpus were ligated [20]. Following ligation of the pylorus and forestomach, severe hemorrhagic damage developed in the proximal 3 cm of the esophagus in a time-dependent manner as assigned of gastritis induction by this model. Animals were sacrificed and the stomachs were autopsied 4 h after the double ligation to examine the protective effect of standard drug and GSS ethanolic extract.

#### 2.6.4 Experimental design

Twenty-five rats were divided into five groups (n = 5). Group I was the normal healthy group that was subjected to sham operation without pylorus ligation, serving as the SH-OP group. Groups II, III, IV&V were exposed to pyloric ligation operation to induce gastritis. Group II served as the positive control group with pyloric ligation surgery and was known as the PYL-LIG group. Group III received omeprazole at a dose of 20 mg/kg along with pyloric ligation. Omeprazole is an acid secretion suppressor that specifically inhibits the H^+^/K^+^ ATPase system, and this group was designated as the reference group, referred to as the PYL-LIG omep group. Groups IV &V were treated with GSS ethanolic extract in two doses 200 and 400 mg/kg respectively in conjunction with pyloric ligation. These two groups were labeled as PYL-LIG GSS 200 mg/kg and PYL-LIG GSS 400 mg/kg.

#### 2.6.5 Tissue collection and sample preparation

The animals were euthanized by decapitation. The stomach of each animal was removed and rinsed with chilled phosphate buffer saline to eliminate any blood clots. The stomach tissues were dried and fixed on paraffin cardboard for further analysis. Each stomach tissue was divided into two equal portions. One portion of each animal`s stomach tissue was immediately placed in a 10 % (v/v) formal saline solution for histopathological and immunohistochemical evaluations. The other portion was homogenized and used for biochemical and immunoblotting assessments.

The homogenate was prepared in 0.05 M phosphate buffer (pH 7) using a polytron homogenizer at 4 °C. Subsequently, the homogenate was centrifuged at 10,000 rpm for 20 min to remove the cell debris, unbroken cells, nuclei, erythrocytes, and mitochondria. The supernatant (cytoplasmic extract) was analyzed using ELISA to measure various biochemical parameters such as SOD, IL-6, ROS, and TNF-*α*. Additionally, Real-Time Polymerase Chain Reaction (RT-PCR) was utilized to determine the gene expression of iNOS, and IκB*α*.

#### 2.6.6 Assessment of oxidative stress, antioxidant activity: inflammatory biomarkers (ROS, SOD, TNF-*α*, and IL-6)

ALL ELISA kits underwent quantification via the employment of an ELISA reader. Utilizing an Enzyme-Linked Immuno-Sorbent Assay (ELISA) plate reader (Stat Fax 2200, Awareness Technologies, Florida, USA), color absorbance within the optical-density (OD) range of 490–630 nm was assessed. The determination of protein content in the tissue was conducted in accordance with the Bradford et al. method [21], utilizing the Genei, Bangalore, protein estimation kit.

#### 2.6.7 Determination of iNOS, and IκB*α* expression by RT-PCR

Total RNA was extracted from all the samples included in the study using the Direct-zol RNA Miniprep Plus kit (Catalog # R2072, ZYMO RESEARCH CORP. USA). The quantity and quality of the extracted RNA were assessed using a Beckman dual spectrophotometer (USA). For reverse transcription of the extracted RNA followed by PCR in a single step, we employed the SuperScript IV One-Step RT-PCR kit (Catalog # 12594100, Thermo Fisher Scientific, Waltham, MA USA). The thermal profile used for this process included an initial reverse transcription (RT) step at 55°C for 10 minutes, followed by RT enzyme inactivation at 95°C for 2 minutes. Subsequently, the amplification process consisted of denaturation at 95°C for 10 seconds, annealing at 55°C for 10 seconds, and extension at 72°C for 30 seconds. This amplification cycle was repeated 40 times, and a single step of the final extension was performed at 72°C for 5 minutes. The primer sequences for the targeted genes can be found in Table 1. After the RT-PCR, the data were expressed in terms of Cycle threshold (Ct). The PCR data sheet contained Ct values for the assessed genes (iNOS, and IκB*α*) in relation to the corresponding housekeeping gene (GAPDH).To measure the gene expression of specific genes, it's essential to use a control sample. The Relative Quantification (RQ) of each target gene was quantified and normalized to the housekeeping gene using the delta-delta Ct (ΔΔCt) calculation. The RQ of each gene was calculated by applying the formula 2^(-∆∆Ct)^.

**Table 1:** Primers for quantitative real-time PCR.

Genes	Primer sequence	Gene bank accession number
iNOS	F:TTGTGGCACACTTGTTCAACCTGG R:TCACACGCATACAAGACCACAGGA	NM_001004216.1
IκB*α*	F: GCTGGGGTTACTGAGTGC R: CCTGTACATGACCCCAGTGGCT	AY547379.1
GAPDH	F: GACATCAAGAAGGTGGTGAAGCAG R: GACATCAAGAAGGTGGTGAAGCAG	NG_028301.2

#### 2.6.8 LD50 study

Sprague Dawley rats were given GSS ethanolic extract at fixed dosages of 5, 50, 300, and 2000 mg/kg. A sighting study was used to establish the first dose, which was expected to generate observable toxicity but not severe effects or fatality. Subsequent dosages were changed in response to observed indicators of toxicity or fatality, and this process was repeated until the dose that caused obvious harm or no effects at the highest dose was determined. GSS ethanolic extract was supplied as a single dosage via oral gavage through a stomach tube after the rats had fasted prior to dosing. Each dose was tested on five rats. Observations included daily clinical monitoring, weekly body weight assessments, and a gross necropsy [22].

#### 2.6.9 Histopathological evaluation

In a routine manner, stomach specimens that had been fixed with formalin were subjected to dehydration using a series of alcohol concentrations, followed by clearing in xylol and subsequent embedding in paraffin. Serial sections measuring 4–5 micrometers in thickness were obtained from the paraffin blocks that had been prepared in advance. These sections were subsequently stained using the Hematoxylin and Eosin (H&E) technique [23]. The images were captured utilizing light microscopy equipment manufactured by Olympus in Japan.

#### 2.6.10 Immunohistochemical evaluation

The paraffin-embedded rat stomach tissues, which had been corrected for antigens, were subjected to deparaffinization. Subsequently, they were incubated overnight at a temperature of 4°C with a primary anti-COX-2 antibody (Abcam, Cambridge, MA, USA, ab31163) at a dilution of 1:100. Subsequently, the transparencies were subjected to incubation with a secondary antibody specific to rats, which was coupled to horseradish peroxidase. Following this, the transparencies were counterstained with hematoxylin.

### 2.7 Molecular modeling

Computer-guided docking experiments were carried out using AutoDock Vina [24]. Seven ligands were selected according to the result of UHPLC-QTOF-MS/MS of GSS ethanolic extract representing the significant compounds found in it. Human interleukin-6 (IL-6) crystal structures (protein database (PDB) code:1ALU) [25], Superoxide Dismutase (SOD) (PDB code: 1CB4) [26], human interleukin-10 (IL-10) (PDB code: 2H24) [27], Heme-oxygenase-1(HO-1) (PDB code: 2PRG) [28] and Kelch-like ECH-associated protein 1 (Keap-1) (PDB code: 7OFE) [29] were retrieved from the RCSB Protein Data Bank website (https://www.rcsb.org/) [30]. The structures of the compounds synthesized were drawn using the ChemOffice tool (ChemDraw 16.0) assigned with proper 2D orientation [31]. The energy of each molecule was minimized using ChemBio3D and was then used as input for AutoDock Vina, to carry out the docking simulation. For each protein structure, the binding sites were predicted using the binding site finder of the molecular operating environment (MOE) tool and CASTp analysis [32]. The protein preparation was done using the reported standard protocol by removing the co-crystallized ligand, water molecules, and cofactors; the target protein file was prepared by leaving the associated residue with protein using Auto preparation of target protein file AutoDock 4.2 (MGLTools 1.5.6). The graphical user interface program was used to set the grid box for docking simulations. The grid was set so that it surrounds the region of interest in the macromolecule. The docking algorithm provided with AutoDock Vina v.1.2.0 was used to search for the best-docked conformation between ligand and protein. During the docking process, a maximum of nine conformers were considered for each ligand. Then, the resulting docking poses were visually examined with BIOVIA Discovery Studio, and interactions with binding pocket residues were studied. Poses fitting into the binding pocket with the top scores and showing useful ligand enzyme contacts were selected [33].

### 2.8 Statistical analysis

We analyzed the data and computed several metrics, including percentages, numerical counts, mean, and standard deviation (SD) for each category. To make comparisons between various groups and enhance our comprehension of the data, we employed a statistical technique known as one-way analysis of variance (ANOVA). Following the ANOVA examination, we conducted a Tukey post hoc analysis to delve deeper into the outcomes. The entire data analysis process was executed using GraphPad Prism software (version 8.00). Statistical significance was determined with a threshold of p-value below 0.05.

## 3 Results

### 3.1 UHPLC-QTOF-MS/MS profiling of *Gliricidia sepium* (Jacq.) Kunth. ex. Walp. stem ethanolic extract

Metabolic profiling of the crude ethanolic GSS extract was demonstrated using UHPLC in the negative mode. The processed data set was subjected to molecular formula prediction and peak identification via dereplication using ChemCalc online, Dictionary of Natural Products (DNP) database, Global Natural Product Social Molecular Networking (GNPS) database, Human Metabolome Database (HMDB), MassBank of North America (MoNA) database, MassBank Europe Mass Spectral Database, food database, Yeast Metabolome Database (YMDB), SIRIUS software, and MZmine software.

Dereplication and chemotaxonomic classification result in the identification of 23 compounds in the crude ethanolic GSS extract that belongs to various classes including flavonoids, fatty acids and their derivatives, triterpenoid saponins, and phenolic acids as shown in Table 2, Fig. 1, and Figs. S1 to S23.

**Table 2:** UHPLC-QTOF-MS/MS Profiling of *Gliricidia sepium* (Jacq.) Kunth. ex. Walp. stem ethanolic extract.

	M	[M − H]^−^ *m*/*z*	ppm error	Compound Name	Molecular formula	Rt (min)	MS/MS Fragmentation	Reference(s)	Compound class
1.	176.17	175.0607	0.87	2-Isopropyl malic acid	C_7_H_12_O_5_	0.81	175.0607,157.0491 115.0397,113.0601 85.0655	[34]	Fatty acid derivatives
3.	164.16	163.0396	0.50	4-Hydroxycinnamic acid	C_9_H_8_O_3_	2.15	163.4545,119.0500 93.0342	[35]	Phenolic acids
4.	740.7	739.2086	0.06	Kaempferol−3-*O*-robinoside−7-*O*-rhamnoside	C_33_H_40_O_19_	2.53	739.2047,593.1512 285.0403,284.0326 255.0303	[36]	Flavonoids
5.	166.17	165.0554	1.40	2-Hydroxyhydrocinnamic acid	C_9_H_10_O_3_	2.56	165.0573,147.0448 121.0663	[37]	Phenolic acids
7.	188.22	187.0973	1.42	Azelaic acid	C_9_H_16_O_4_	2.81	187.0975,143.1078 125.0964,123.0801 97.0653	[38]	Fatty acid derivatives
8.	464.4	463.0895	3.99	Isoquercitrin	C_21_H_20_O_12_	2.82	463.1242,301.0347 300.0265,271.0237 255.0287	[39]	Flavonoids
9.	594.5	593.1511	0.77	Kaempferol−3-*O*-rutinoside	C_27_H_30_O_15_	2.83	593.1500,285.0403 284.0326,255.0297	[40]	Flavonoids
10.	432.4	431.0979	0.18	Apigenin −7-*O*-glucoside	C_21_H_20_O_10_	3.08	431.0970,269.0449 268.0377	[41]	Flavonoids
11.	432.4	431.0979	0.18	Kaempferol−7-*O*-rhamnoside	C_21_H_20_O_10_	3.49	431.0945,285.0395 284.0324	[42]	Flavonoids
12.	286.24	285.0401	0.66	Luteolin	C_15_H_10_O_6_	3.55	285.0400,151.0030 133.0291	[43]	Flavonoids
13.	270.24	269.0452	0.75	Apigenin	C_15_H_10_O_5_	3.86	269.0442,151.0025 117.0335	[44]	Flavonoids
14.	330.5	329.2331	0.91	9,12,13-Trihydroxyoctadec−10-enoic acid	C_18_H_34_O_5_	3.94	329.2336,311.2220 293.2105,229.1438 199.1338,171.1018 129.0919	[45]	Fatty acid derivatives
15.	256.25	255.0658	0.26	Liquiritigenin	C_15_H_12_O_4_	4.12	255.0655,135.0081 119.0501,91.00183	[46]	Flavonoids
16.	913.1	911.5003	−0.14	Soyasaponin II	C_47_H_76_O_17_	4.20	911.5001,765.4468 615.3914,457.3652	[47]	Triterpenoid saponins
17.	943.1	941.5114	0.43	Soyasaponin I	C_48_H_78_O_18_	4.22	941.5150,923.5018 633.4006,615.3909 457.3698	[47]	Triterpenoid saponins
18.	797	795.4530	−0.10	Soyasaponin III	C_42_H_68_O_14_	4.30	795.4561,615.3904 457.3702	[48]	Triterpenoid saponins
19.	927.1	925.5163	0.24	Kaikasaponin III	C_48_H_78_O_17_	4.31	925.5190,779.4573 617.4012,599.3934 441.3712	[49]	Triterpenoid saponins
20.	314.5	313.2381	0.69	9,10-Dihydroxyoctadec−12-enoic acid	C_18_H_34_O_4_	4.60	313.2383,277.2185 201.1129,171.1020	[50]	Fatty acid derivatives
21.	316.5	315.2537	0.52	9,10-Dihydroxyoctadecanoic acid	C_18_H_36_O_4_	4.80	315.2535,297.2425 201.1115,171.1024 127.1123	[51]	Fatty acid derivatives
22.	294.4	293.2115	−0.58	13-Hydroxyoctadeca−9,11,15-trienoic acid	C_18_H_30_O_3_	4.94	293.2134,275.2011 223.1331,195.1381 183.1378	[52]	Fatty acid derivatives
23.	296.4	295.2276	0.95	9-Hydroxy−10,12-octadecadienoic acid	C_18_H_32_O_3_	5.13	295.2258,277.2153 171.1009	[45]	Fatty acid derivatives
24.	294.4	293.2115	−0.58	13-Oxooctadeca−9,11-dienoic acid	C_18_H_30_O_3_	5.33	293.2109,249.2198 167.1068,113.0968	[53]	Fatty acid derivatives
25.	272.42	271.2276	1.03	16-Hydroxyhexadecanoic acid	C_16_H_32_O_3_	5.66	271.2273,253.2169 225.2227	[54]	Fatty acid derivatives

**Fig. 1:**
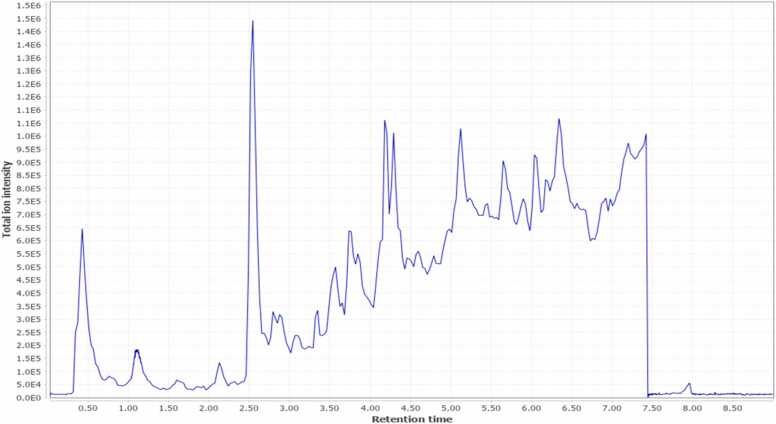
Total ion chromatogram of UHPLC-QTOF-MS/MS Profiling in the negative mode of *Gliricidia sepium* (Jacq.) Kunth. ex. Walp. stem ethanolic extract.

Eight flavonoids were identified including kaempferol-3-*O*-robinoside-7-*O*-rhamnoside, isoquercitrin,kaempferol-3-*O*-rutinoside,apigenin-7-glucoside,kaempeferol-7-*O*-rhamnoside,luteolin, apigenin, and liquiritigenin. Furthermore, two phenolic acids were detected including 4-hydroxycinnamic acid and 2-hydroxyhydrocinnamic acid.

On the other hand, four triterpenoid saponins were found including soyasaponin I, soyasaponin II, soyasaponin III, and kaikasaponin III. Moreover, nine fatty acids derivatives were dereplicated including 2-isopropyl malic acid, azelaic acid, 9,12,13-trihydroxyoctadec-10-enoic acid, 9,10-dihydroxyoctadec-12-enoic acid, 9,10-dihydroxyoctadecanoic acid, 13-hydroxyoctadeca-9,11,15-trienoic acid, 9-hydroxy-10,12-octadecadienoic acid, 13-oxooctadeca-9,11-dienoic acid, and 16-hydroxyhexadecanoic acid.

The major compounds identified in the GSS ethanolic extract are kaempferol-3-*O*-robinoside-7-*O*-rhamnoside, soyasaponin III, soyasaponin I, 9-hydroxy-10,12-octadecadienoic acid, 9,12,13-trihydroxy octadec-10-enoic acid, and16-hydroxyhexadecanoic acid.

### 3.2 Molecular networking

**Fig. 2:**
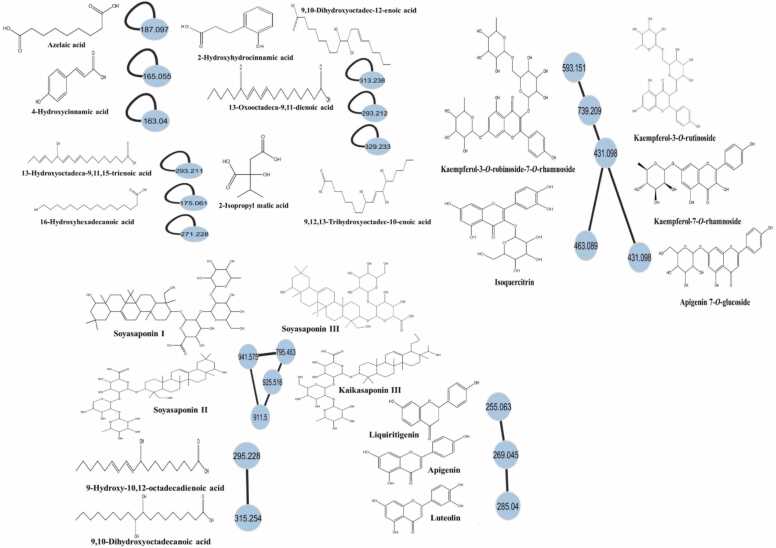
Molecular networking using UHPLC-QTOF- MS/MS data for *Gliricidia sepium* (Jacq.) Kunth. ex. Walp. stem ethanolic extract in negative mode.

The molecular networking analysis for ethanolic extract of GSS showed that compounds identified previously with UHPLC-QTOF-MS/MS in the negative mode formed clusters with each other, while others did not. The fragmentation records were organized by molecular networking and successively dereplicated. In the molecular network generated, the MS/MS spectra comprised 4 clusters, as shown in Fig. 2. Saponins including soyasaponin II, soyasaponin I soyasaponin III, and kaikasaponin III formed one cluster. Flavonoids formed another cluster including liquiritigenin, luteolin, and apigenin. Isoquercitrin, apigenin-7-*O*-glucoside,kaempferol-3-*O*-rutinoside,kaempferol-7-*O*-rhamnoside,and kaempferol-3-*O*-robinoside-7-*O*-rhamnoside formed another cluster. 9,10-dihydroxyoctadecanoic acid and 9-hydroxy-10,12-octadecadienoic acid formed another cluster. The remaining compounds did not form clusters such as 2-hydroxyhydro cinnamic acid, 9,12,13-trihydroxyoctadec-10-enoic acid, 16-hydroxyhexadecanoic acid, 9,10-dihydroxyoctadec-12-enoic acid, 13-oxooctadeca-9,11-dienoic acid, 4-hydroxycinnamic acid, 13-hydroxyoctadeca-9,11,15-trienoic acid, 2-isopropyl malic acid, and azelaic acid.

### 3.3 Total phenolic and flavonoid contents of *Gliricidia sepium* (Jacq.) Kunth. ex. Walp. stem ethanolic extract

The total phenolic content of GSS ethanolic extract was 38.78 ± 1.609 µg of gallic acid equivalent/mg (Table 3). Similarly, the total flavonoid content was 5.62 ± 0.50 µg of Rutin equivalent/mg (Table 3).

**Table 3:** Total phenolic and flavonoid contents of *Gliricidia sepium* (Jacq.) Kunth. ex. Walp. stem ethanolic extract.

Total phenolic content (µg gallic acid equivalent/mg)	38.78 ± 1.609
Total flavonoid content (µg Rutin equivalent/mg)	5.62 ± 0.50

*The average of the readings of the 3 replicates was taken.

### 3.4 Biological results

#### 3.4.1 LD50 result

*Gliricidia sepium* (Jacq.) Kunth. ex. Walp. stem ethanolic extract was administered orally to Sprague Dawley rats at increasing doses up to 2000 mg/kg body weight. No toxic effects or deaths were recorded within 24 hours of administration. This indicates that the median lethal dose (LD50) for *Gliricidia sepium* (Jacq.) Kunth. ex. Walp. stem ethanolic extract in rats is probably above 2000 mg/kg. Prior literature states that substances with LD50 values over 50 mg/kg body weight are typically regarded as non-toxic [22].

#### 3.4.2 Macroscopical findings

**Fig. 3:**
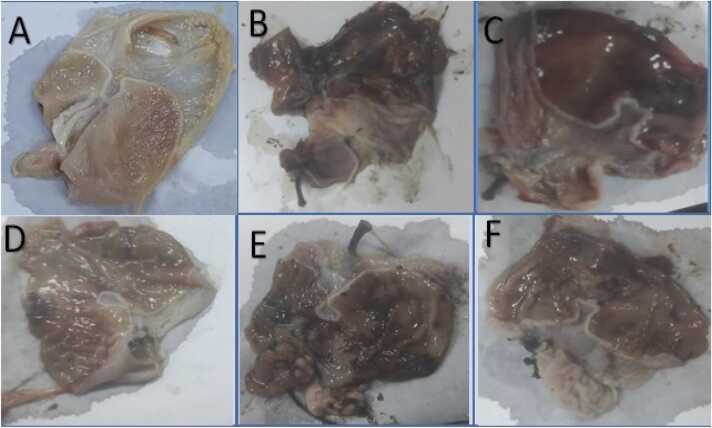
Macroscopic examination of gastric tissues of rat where A, a photomicrograph of the gastric tissue of the normal healthy group was subjected to sham operation without pylorus ligation, serving as the SH-OP group; B&C, a photomicrograph of the gastric tissues of the positive control group with pyloric ligation surgery and known as PYL-LIG group; D, a photomicrograph of the gastric tissue from the group that received omeprazole at a dose of 20 mg/kg along with pyloric ligation, designated as the reference group, and referred to as the PYL-LIG omep group; E, a photomicrograph of the gastric tissue from the group that was treated with GSS ethanolic extract at a dose of 200 mg/kg in conjunction with pyloric ligation, labeled as the PYL-LIG GSS 200 mg/kg group and F, a photomicrograph of the gastric tissue of the group which received GSS extract in dose 400 mg/kg with pyloric ligation, identified as PYL-LIG GSS 400 mg/kg.SH-OP; Sham Operation group, PYL-LIG; Pyloric Ligation group, PYL-LIG omep; Pyloric Ligation omeprazole group, PYL-LIG GSS 200 mg/kg; Pyloric Ligation with *Gliricidia sepium* (Jacq.) Kunth. ex. Walp. stem ethanolic extract at dose 200 mg/kg, PYL-LIG GSS 400 mg/kg; Pyloric Ligation with *Gliricidia sepium* (Jacq.) Kunth. ex. Walp. stem ethanolic extract at a dose of 400 mg/kg.

During the macroscopic examination of the gastric tissue from the normal control group (Fig. 3A), there were no signs of hemorrhagic lesions or mucosal erosions. However, in the positive group (Fig. 3B & C), there was a notable increase in gastric lesions compared to the normal control group. Conversely, the groups treated with omeprazole (20 mg/kg), GSS ethanolic extract (200 mg/kg), and GSS ethanolic extract (400 mg/kg) (Fig. 3D, E, and F, respectively) exhibited minimal mucosal injury, with only superficial disruption observed in the gastric epithelial lining. However, the observed treatment effect of both GSS ethanolic extract groups was lower than the superior effect achieved by the omeprazole.

#### 3.4.3 Assessment of oxidative stress and antioxidant biomarkers (ROS and SOD)

**Fig. 4:**
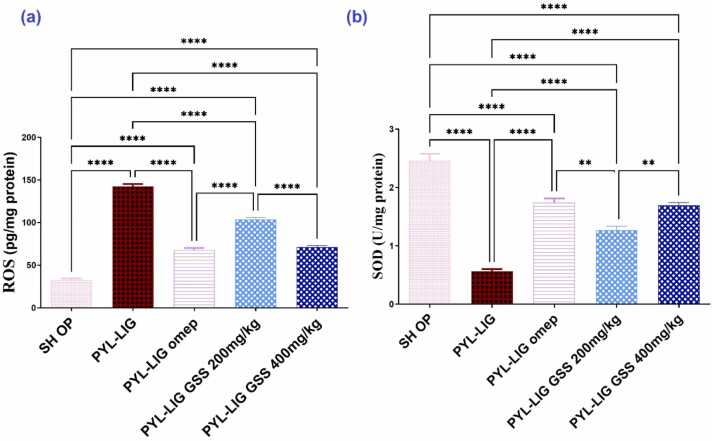
Effect of *Gliricidia sepium* (Jacq.) Kunth. ex. Walp. stem ethanolic extract (200 & 400 mg/kg) on ROS (a), SOD (b) under pyloric ligation-induced gastritis at (n = 5). Statistical analysis was performed using the one-way analysis of variance (ANOVA) followed by Tukey’s multiple comparison test. ROS; reactive oxygen species, SOD; superoxide dismutase. SH OP; sham operation group, PYL-LIG; pyloric ligation group, PYL-LIG omep; pyloric ligation omeprazole group, PYL-LIG GSS 200 mg/kg; pyloric ligation *Gliricidia sepium* (Jacq.) Kunth. ex. Walp. stem ethanolic extract group at dose 200 mg/kg, PYL-LIG GSS 400 mg/kg; pyloric ligation *Gliricidia sepium* (Jacq.) Kunth. ex. Walp. stem ethanolic extract group at a dose of 400 mg/kg. * * p ≤ 0.01, * ** * P ≤ 0.0001.

In comparison to the SH OP group, the PYL-LIG group had a substantial *(p* < *0.0001)* rise in ROS levels by 4.38-folds with a significant *(p* < *0.0001)* decrease in SOD activity by 77.15 %. When compared to the PYL-LIG group, the PYL-LIG omep (20 mg/kg) treated group significantly *(p* < *0.0001)* lowered the ROS level by 2.1-folds and significantly *(p* < *0.0001)* increased SOD activity by 47.77 %. The oral administration of GSS to Groups (IV and V) showed a considerable *(p* < *0.0001)* decrease in ROS by 1.38-fold and 2.00-fold, respectively. On the other hand, SOD activities significantly *(p* < *0.0001)* increased by 28.59 % and 46.07 %, respectively in comparison to the PYL-LIG group. as shown in Fig. 4(a, b).

A notable significance (*p* < *0.0001)* in ROS and (*p* < *0.01)* in SOD was recorded by administration of 200 vs 400 mg GSS to the treated groups. The effect of GSS ethanolic extract at a dose of 400 mg/kg is the same effect of the omeprazole group at a dose of 20 mg/kg on ROS, SOD concentrations when compared to each other. The results are represented in Fig. 4.

#### 3.4.4 Assessment of anti-inflammatory biomarkers (TNF-*α* and IL-6)

Pyloric ligation caused a substantial *(p* < *0.0001)* elevation in TNF-*α,* and IL-6 levels by 4.39 and 3.76-folds, respectively as shown in PYL-LIG when compared by sham group. by the administration of omeprazole at a dose of 20 mg/kg, a substantial *(p* < *0.0001)* decrease was recorded in the levels of TNF-*α*, and IL-6 by 258.70 % and 200.24 %, respectively in comparison to the PYL-LIG group.

The administration of GSS at dose 200 mg/kg showed a considerable *(p* < *0.0001)* decrease in TNF-*α*, and IL-6 levels by 164.41 % and 111.38 % respectively as compared to PYL-LIG group but this action is still a little *(p* < *0.0001)* improvement in inflammatory status when compared to the other treatments (PYL-LIG omep and PYL-LIG GSS 400 mg/kg).

**Fig. 5:**
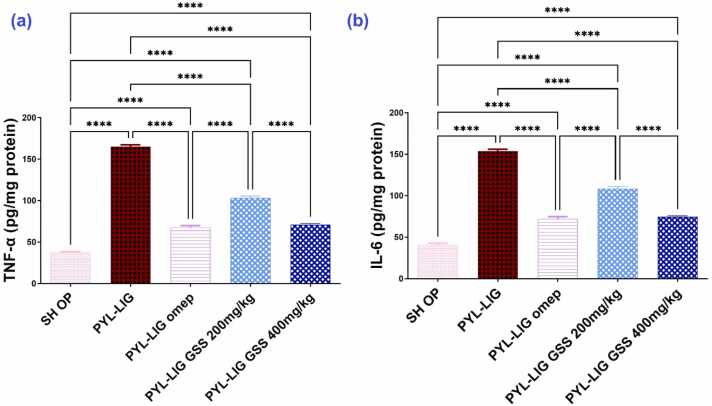
Effect of *Gliricidia sepium* (Jacq.) Kunth. ex. Walp. stem ethanolic extract (200 & 400 mg/kg) on TNF-*α* (a), and IL-6 (b) under pyloric ligation-induced gastritis at (n = 5). Statistical analysis was performed using the one-way analysis of variance (ANOVA) followed by Tukey’s multiple comparison test. TNF-*α*; tumor necrosis factor alpha, IL-6; interleukin-6. SH OP; sham operation group, PYL-LIG; pyloric ligation group, PYL-LIG omep; pyloric ligation omeprazole group, PYL-LIG GSS 200 mg/kg; pyloric ligation *Gliricidia sepium* (Jacq.) Kunth. ex. Walp. stem ethanolic extract group at dose 200 mg/kg, PYL-LIG GSS 400 mg/kg; pyloric ligation *Gliricidia sepium* (Jacq.) Kunth. ex. Walp. stem ethanolic extract group at a dose of 400 mg/kg. * ** * P ≤ 0.0001.

Conversely, the effect of GSS ethanolic extract at a dose of 400 mg/kg is nearly the same effect of the omeprazole group at a dose of 20 mg/kg without any notable significance. where, the results revealed a crucial decline in TNF-*α*, and IL-6 levels by 250.07 % and 194.37 % when compared to the PYL-LIG group. The results are represented in Fig. 5(a and b).

#### 3.4.5 The gene expression of iNOS and IκB*α*

**Fig. 6:**
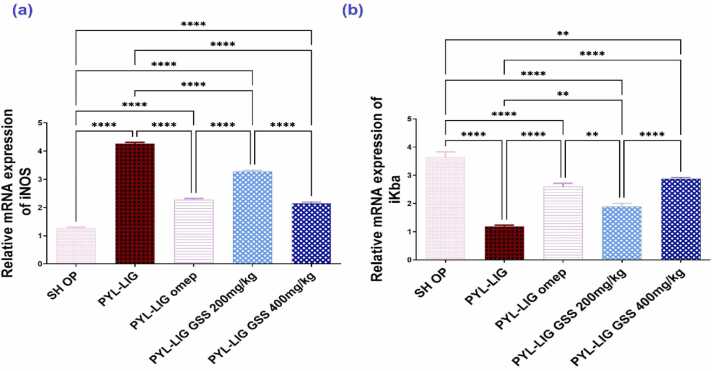
Effect of *Gliricidia sepium* (Jacq.) Kunth. ex. Walp. stem ethanolic extract (200 & 400 mg/kg) on iNOS (a) and IκB*α* (b) under pyloric ligation-induced gastritis at (n = 5). iNOS; Inducible nitric oxide synthase, IκB*α*; inhibitor of nuclear factor kappa B. SH OP; sham operation group, PYL-LIG; pyloric ligation group, PYL-LIG omep; pyloric ligation omeprazole group, PYL-LIG GSS 200 mg/kg; pyloric ligation *Gliricidia sepium* (Jacq.) Kunth. ex. Walp. stem ethanolic extract group at dose 200 mg/kg, PYL-LIG GSS 400 mg/kg; pyloric ligation *Gliricidia sepium* (Jacq.) Kunth. ex. Walp. stem ethanolic extract group at a dose of 400 mg/kg. Statistical analysis was performed using the one-way analysis of variance (ANOVA) followed by Tukey’s multiple comparison test. ** p ≤ 0.01,*​​​​​*** P ≤ 0.0001.

The PYL-LIG group exhibited a significant increase in iNOS expression by 3.38 folds and a decrease in IκB*α* levels by 3.05 folds compared to the SH OP group. In contrast, the PYL-LIG omep (20 mg/kg) group showed a substantial reduction in iNOS expression by 1.88 folds and an increase in IκB*α* levels by 2.18 folds compared to the PYL-LIG group. The oral PYL-LIG GSS at a dose of 200 mg/kg significantly raised iNOS expression by 1.30 folds and lowered IκB*α* levels by 1.59 folds when compared to the PYL-LIG omep (20 mg/kg) group. Notably, the oral PYL-LIG GSS at a dose of 400 mg/kg significantly lowered iNOS expression by 1.99 folds and raised IκB*α* levels by 2.41 folds compared to the PYL-LIG GSS (200 mg/kg) group. The expression of iNOS and IκB*α* in response to the GSS ethanolic extract at 400 mg/kg was similar to that observed with omeprazole at 20 mg/kg, indicating comparable effects. The results are shown in Fig. 6.

#### 3.4.6 Histopathological evaluation

**Fig. 7:**
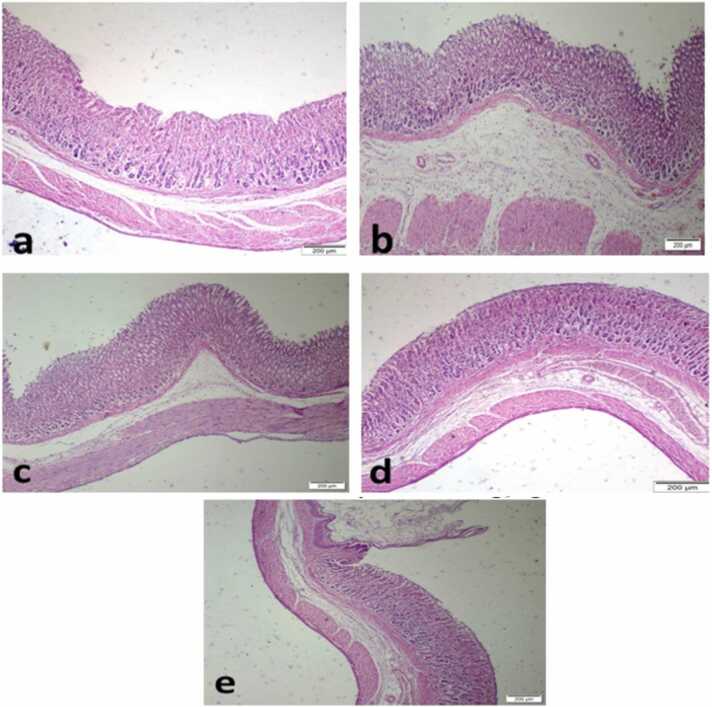
Representative histopathological images of H&E stained gastric tissue. a: SH OP normal group which was subjected to sham operation without pylorus ligation and showed normal histological architecture of gastric regions (mucosa, submucosa, musculasa, and serosa). b: The PYL-LIG group served as the positive control group with pyloric ligation surgery which revealed inflammatory cell infiltration in the mucosal layer, and necrosis of gastric mucosal cells with cystic dilatations. c: PYL-LIG Omep is the reference drug-treated group with pyloric ligation and treated with omeprazole at a dose of 20 mg/kg showed moderate inflammatory cell infiltration mixed with moderate edema in the submucosal layer. d &e: PYL-LIG GSS treated groups which receive *Gliricidia sepium* stem (Jacq.) Kunth. ex. Walp. stem ethanolic extract in two doses of 200&400 mg/kg respectively with pyloric ligation exhibited mild infiltration of inflammatory cells mixed with mild edema. (Scale bar, 50 µm).

Fig. 7 depicts histological changes observed in the stomach tissue of different experimental groups. The typical histological structure of different stomach regions, including the mucosa, submucosa, muscularis, and serosa, was observed by the study of gastric sections obtained from healthy rats (Fig. 7a) under a light microscope. In contrast, the histological sections of rats stimulated with PYL-LIG had significant histopathological damage. The presence of focal necrosis of the gastric mucosa, erosion, submucosal edema, severe diffuse inflammatory cell infiltration, and dilated blood vessels was observed, as depicted in Fig. 7b. In contrast, the administration of omeprazole at a dosage of 20 mg/Kg, which served as a reference drug, resulted in notable amelioration of the gastric tissue condition when compared to the control group. Specifically, the examination of the stomachs in this group revealed a mild alteration in the mucosal layer, along with a moderate infiltration of inflammatory cells and a moderate diffuse edema in the submucosal layer (Fig. 7c). The experimental groups (GSS) exhibited a statistically significant improvement effect (Fig. 7d &e) in comparison to the control positive group. However, the observed treatment effect of both GSS ethanolic extract groups was lower than the superior effect achieved by the reference medication.

#### 3.4.7 COX-2 protein expression in different treated groups

**Fig. 8:**
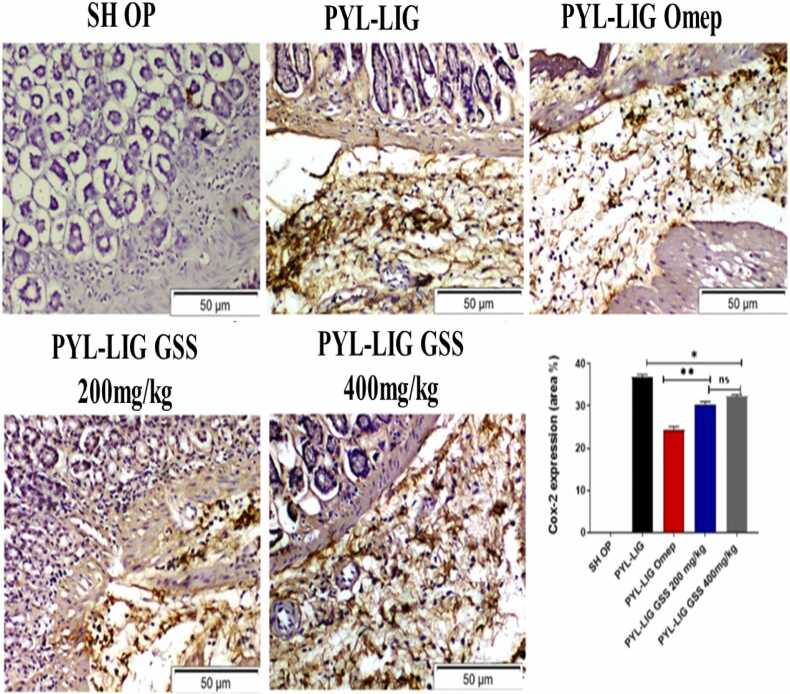
Immunohistochemical staining of COX-2 in the gastric tissue. The quantitative data are provided as mean ± S.E. of three gastritis areas from three different animals (n = 3), P < 0.05 * : when compared to the normal. * *: when compared to the Eth-induced group. Ns: non-significance.

The results of the immunohistochemistry study indicate that rats exposed to PYL-LIG exhibited a significant upregulation in the gene expression of COX-2 (p < 0.05). Conversely, rats treated with omeprazole had a significant decrease in COX-2 expression (p < 0.05) (see Fig. 8). The groups that were treated with GSS ethanolic extract exhibited a reduction in protein expression in comparison to the control group.

### 3.5 Molecular docking

A molecular docking study was carried out to find out the interaction between GSS ethanolic extract and the target receptors. In total, 30 docking analyses were carried out. Their free energy binding is shown in Table 4. The best molecular interaction based on the molecular interaction is represented in Table 5. Soyasaponin I was most frequently interacting with proteins.

**Table 4:** Free binding energy interaction of *Gliricidia sepium* (Jacq.) Kunth. ex. Walp. stem ethanolic extract with the target proteins.

Ligand	Free energy of binding (kcal/mol) Target proteins				
	IL-6(PDB code: 1ALU)	SOD(PDB code: 1CB4)	IL-10(PDB code: 2H24)	HO-1 (PDB code:2PRG)	Keap-1 (PDB code: 7OFE)
kaempferol−3-*O*-robinoside−7-*O*-rhamnoside	−18.38	−16.78	−12.067	−15.20	−19.62
soyasaponin III 4	−6.08	−12.65	−11.65	−9.68	−13.02
soyasaponin I 5	−20.70	−18.62	−13.72	−16.94	−20.36
9-hydroxy−10,12-octadecadienoic acid (2)	−6.17	−12.94	−8.92	−11.62	−10.71
9,12,13-trihydroxy octadec−10-enoic acid (1)	6.13	−11.55	−9.41	−11.42	−12.34
16-hydroxyhexadecanoic acid(3)	−6.03	−11.67	−9.33	−11.80	−10.42

**Table 5:** Summary of Molecular docking results for *Gliricidia sepium* (Jacq.) Kunth. ex. Walp. stem ethanolic extract with target proteins.

Ligands	Hydrogen bonds between atoms of ligands and amino acids of receptor						S- score (binding energy) (kcal/mol)
	ligands Atoms	Receptor		Type	Distance (Å)	Energy (kcal/mol)	
		Atoms	Residues				
Human Interleukin−6 crystal structures (PDB code: 1ALU)							
kaempferol−3-*O*-robinoside−7-*O*-rhamnoside	O48	OE2	Glu 95	H-donor	2.45	−1.2	−18.38
	O27	ND2	Asn144	H-receptor	2.83	−3.6	
	O28	CB	Asn 144	H-acceptor	2.44	−3.9	
soyasaponin I	O64	OG	Ser176	H-donor	3.10	−3.6	−20.70
	O62	CA	Ala68	H-acceptor	2.69	−1.0	
Superoxide Dismutase (PDB code: 1CB4)							
kaempferol−3-*O*-robinoside−7-*O*-rhamnoside	O39	O	Arg141	H-donor	1.98	−1.0	−16.78
	O51	OD1	Asn51	H-donor	2.53	−0.6	
	O35	N	Cys55	H-acceptor	2.66	−3.2	
soyasaponin I	O61	O	Thr52	H-donor	2.76	−1.2	−18.62
	O22	NZ	Lys9	H-acceptor	2.66	−5.1	
human interleukin−10 (IL−10) (PDB code: 2H24)							
kaempferol−3-*O*-robinoside−7-*O*-rhamnoside	O55	NE	Arg27	H-acceptor	2.91	−2.8	−12.06
	O54	NE	Arg27	Ionic	2.83	−5.7	
	O54	NH1	Arg27	Ionic	3.88	−0.7	
	O54	NH1	Arg27	Ionic	2.74	−6.4	
	6-ring	CD2	Leu65	Pi-H	3.94	−0.6	
	6-ring	CD2	Leu105	Pi-H	3.86	−0.8	
soyasaponin I	O 67	O	LEU 23	H-donor	3.10	−1.6	−13.72
Heme-oxygenase−1 (PDB code: 2PRG)							
kaempferol−3-*O*-robinoside−7-*O*-rhamnoside	O27	N	Glu343	H-acceptor	2.06	−1.0	−15.20
	O54	CA	Ile281	H-acceptor	2.78	−3.4	
	O41	NE	Arg288	Ionic	2.77	−6.2	
	O41	NH1	Arg288	Ionic	0.67	−7.2	
	O41	NH2	Arg288	Ionic	2.88	−5.3	
soyasaponin I	C11	SD	Met364	H-donor	3.76	−0.9	−16.94
	O63	SD	Met364	H-donor	3.75	−1.1	
	O20	NE	Arg288	H-acceptor	2.78	−3.5	
Kelch-like ECH-associated protein 1 (Keap1) (PDB code: 7OFE)							
kaempferol−3-*O*-robinoside−7-*O*-rhamnoside	O39	OE1	Gln530	H-donor	3.21	−1.2	−19.62
	O8	NH2	Arg415	H-acceptor	2.52	−3.3	
	O49	NE	Arg483	H-acceptor	1.71	−1.3	
	O51	CB	Phe478	H-acceptor	2.87	−1.3	
	O54	NE	Arg415	Ionic	3.68	−1.3	
	O54	NH2	Arg415	Ionic	2.23	−2.3	
	6-ring	6-ring	TYR 525	Pi-pi	3.07	−0.6	
	6-ring	6-ring	Tyr525	Pi-pi	3.82	−0.6	
soyasaponin I	C9	O	Val608	H-donor	3.58	−0.6	−20.36
	O60	O	Val369	H-donor	2.45	−2.4	
	O59	N	Val 369	H-acceptor	2.68	−3.5	

**Fig. 9:**
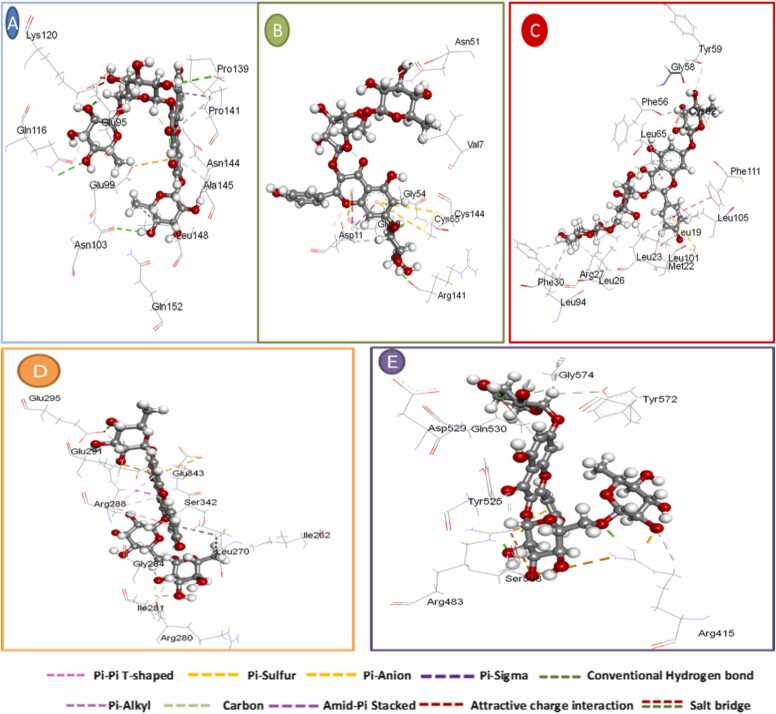
Inter-molecular interactions between A) kaempferol-3-*O*-robinoside-7-*O*-rhamnoside - Human Interleukin-6 B) kaempferol-3-*O*-robinoside-7-*O*-rhamnoside - Superoxide Dismutase C) kaempferol-3-O-robinoside-7-O-rhamnoside – human interleukin-10,D) kaempferol-3-*O*-robinoside-7-*O*-rhamnoside – Heme-oxygenase-1,E) kaempferol-3-*O*-robinoside-7-*O*-rhamnoside - Kelch-like ECH-associated protein 1.

**Fig. 10:**
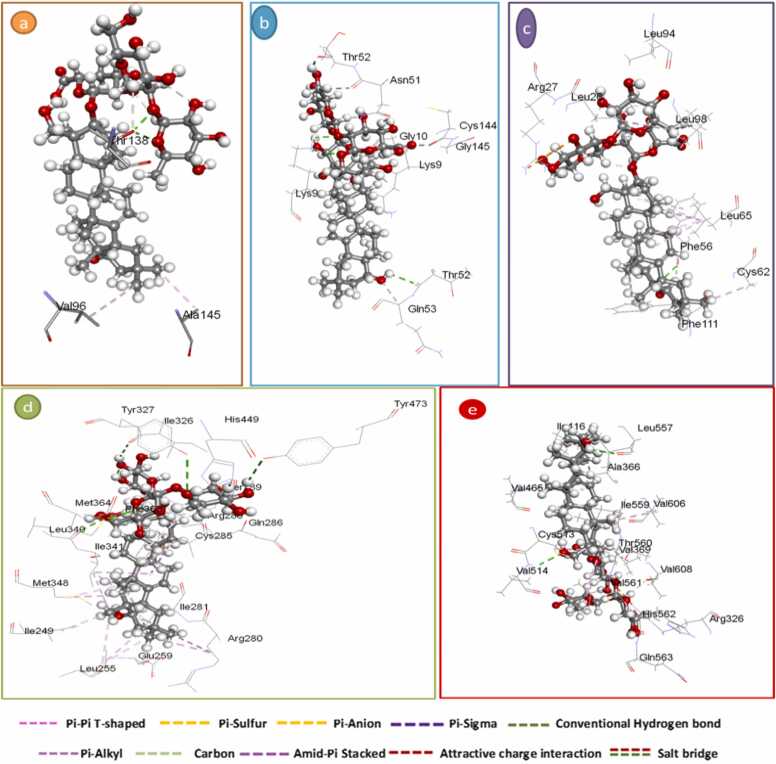
Inter-molecular interactions between a) soyasaponin I - Human Interleukin-6 b) soyasaponin I - Superoxide Dismutase c) soyasaponin I – human interleukin-10,d) soyasaponin I Heme-oxygenase-1,e) soyasaponin I - Kelch-like ECH-associated protein.

There is no co-crystallized ligand for human IL-6 crystal structures (PDB code: 1ALU), so the MOE site finder module was utilized for active pocket prediction. The docked soyasaponin I - human IL-6 complex has established a secured hydrogen bonding interaction with the Ala 68, and Ser 176. It is worth noting that, the oxygen atom of the pyran ring in soyasaponin I has formed a hydrogen bind interaction with Thr 138. In addition, the cyclohexyl ring has formed a Pi-alkyl interaction with Val 96. On the other hand, the docked kaempferol-3-*O*-robinoside-7-*O*-rhamnoside - human interleukin-6 complex has formed hydrogen bonding interaction with Glu 95, Gln 116, Pro 139, and Asn144. The pharmacophoric hot spot residue, Ala 145, formed Pi-alkyl interaction with the cyclohexyl group of soyasaponin I, and the chromene ring of kaempferol-3-*O*-robinoside-7-*O*-rhamnoside (Figs. 9A & 10a).

For SOD (PDB code: 1CB4), There is no co-crystallized ligand, so the MOE site finder module was utilized for active pocket prediction. The oxygen atom of the pyran ring in soyasaponin I exhibited hydrogen bond interaction with Lys 9, and Ser 156. Moreover, the hydroxyl group of the cyclohexane ring showed H-bond with Thr 52. The docked kaempferol-3-*O*-robinoside-7-*O*-rhamnoside showed hydrogen bond interaction with Asn 51, Cys 55, and Arg 141 (Figs. 9B & 10b).

For human IL-10 (PDB code: 2H24), There is no co-crystallized ligand, so the MOE site finder module was utilized for active pocket prediction. Soyasaponin I showed hydrogen bond interaction with Leu 23, and Phe56 with a binding energy score of −13.72 Kcal/mol. Kaempferol-3-*O*-robinoside-7-*O*-rhamnoside showed Hydrogen bonding interaction with Arg 27 with a binding energy score of −12.06 Kcal/mol (Figs. 9C & 10c).

For HO-1 (PDB code: 2PRG), the co-crystallized ligand showed a docking score of −6.92 Kcal/mol. Soyasaponin I showed hydrogen bond interaction Ile326, Tyr327, Leu340, Met 364, and Tyr473 with binding energy score −16.94 Kcal/mol. Kaempferol-3-*O*-robinoside-7-*O*-rhamnoside showed hydrogen bonding interaction with Ser 342 with a binding energy score of −15.20 Kcal/mol. Furthermore, the pharmacophoric hot spot residue Arg 288 has formed hydrogen bonding interaction, and Pi-sigma interaction with both soyasaponin I and kaempferol-3-*O*-robinoside-7-*O*-rhamnoside, respectively (Figs. 9D & 10d).

For Keap-1(PDB code: 7OFE), the co-crystallized ligand showed a docking score of −7.59 Kcal/mol. Soyasaponin I showed hydrogen bond interaction Val 369, Val 514and Leu557 with binding energy score −20.36 Kcal/mol. kaempferol-3-*O*-robinoside-7-*O*-rhamnoside showed hydrogen bonding interaction with Arg415, Arg483, and Gln 530 with binding energy score −19.62 Kcal/mol (Figs. 9E & 10e).

## 4 Discussion

Gastritis is a common gastrointestinal disorder characterized by inflammation of the stomach lining which is affected by oxidative stress, resulting from the imbalance between ROS production and the ability of the body to counter them. During inflammation, phagocytic cells such as macrophages and neutrophils lead to excessive production of ROS that results in activation of antioxidant enzymes such as SOD to regulate the ROS levels and further aggravate inflammation. The exaggerated inflammation due to the excessive production of ROS leads to activating various inflammatory pathways and signaling molecules including TNF-*α* [55]. TNF-*α* is the cytokine that promotes the activation of iNOS, leading to nitric oxide production. Additionally, ROS-induced oxidative stress leads to the degradation of IκBα, a protein that normally inhibits nuclear factor kappa B (NF-κB) [56]. When IκBα is degraded, NF-κB translocates to the nucleus and drives the expression of pro-inflammatory genes, including COX-2. COX-2 facilitates the production of prostaglandins, which perpetuate inflammation and pain [57].

According to our study, the positive control group the PYL-LIG group had a substantial rise in ROS, IL-6, TNF-α, and cox-2 concentrations and significantly reduced SOD levels. These findings align with the previous studies which reported that during inflammation and oxidative stress in gastritis inflammation triggers the upregulation of pro-inflammatory cytokines like TNF-*α*, IL-6, and iNOS which in turn stimulate high levels of ROS production and recruit neutrophils and macrophages [58]. The NF-κB signaling pathway is pivotal in this inflammatory response, as it facilitates the transcription of pro-inflammatory cytokines [59]. Normally, NF-κB remains bound to IκB in the cytoplasm of unstimulated cells. Upon activation by various stimuli, IκB undergoes phosphorylation and degradation, allowing NF-κB to translocate to the nucleus where it promotes the expression of pro-inflammatory genes [60]. Oxidative stress occurs due to an imbalance between oxidants and antioxidants, resulting in excessive ROS generation. This oxidative imbalance stimulates the activity of antioxidant enzymes such as SOD, which play critical roles in neutralizing ROS and products of lipid peroxidation [61].

UHPLC-QTOF-MS/MS Profiling of GSS ethanolic extract revealed the presence of flavonoids, phenolic acids, triterpenoid saponins, and fatty acid derivatives. Kaempferol-3-*O*-robinoside-7-*O*-rhamnoside, soyasaponin III, soyasaponin I, 9-hydroxy-10,12-octadecadienoic acid, 9,12,13-trihydroxyoctadec-10-enoic acid, and 16-hydroxyhexadecanoic acid were the prominent compounds identified in GSS ethanolic extract. Some of the identified compounds were previously isolated from various parts of the *Gliricidia sepium* (Jacq.) Kunth. ex. Walp. stem plant, while others were newly identified for the first time. Kaempferol-3-*O*-robinoside-7-*O*-rhamnoside, kaempferol-3-*O*-rutinoside were previously extracted from the leaves, while isoquercitrin had been isolated from the flowers, and formononetin was earlier found in the heartwood of the stem [8].These major compounds were previously reported to possess antioxidant and anti-inflammatory properties [62], [63], [64], [65].

Kaempferol-3-*O*-robinoside-7-*O*-rhamnoside modulates inflammatory responses by suppressing the expression of TLR2(toll-like receptor 2), TLR4 (toll-like receptor 4), and NF-κB(nuclear factor kappa B) leading to reduced pro-inflammatory cytokine production and downregulating inflammatory enzymes, thereby protecting against ox-LDL-induced inflammatory stress by modulating the TLR-NF-κB signaling pathway [62]. It was also reported that Kaempferol-3-*O*-robinoside-7-*O*-rhamnoside exhibits significant anti-inflammatory and antioxidant activities via reduced expression of inducible iNOS, IL-6, and TNF-*α*[62], [63]. Soyasaponin III and soyasaponin I exhibit anti-inflammatory properties by downregulating MyD88 expression, suppressing TLR4/MyD88 (toll-like receptor 4/myeloid differentiation primary response 88)signaling, and inhibiting NF-κB activation [65]. It was also reported that soyasaponin I possesses anti-inflammatory activity by inhibiting the production of iNOS, IL-6, and TNF-*α*[64].

In our study oral administration of GSS ethanolic extract led to a significant decrease in oxidative stress and inflammatory markers resulting in reduced levels of ROS, IL-6, and TNF-*α*, as well as an increase in SOD concentrations, lowered expression of iNOS and increased expression of IκB*α*. The high dose of GSS ethanolic extract (400 mg/kg) has nearly the same efficacy as omeprazole (20 mg/kg) in mitigating gastritis-related oxidative stress and inflammation. These results underscore the complex interaction between GSS ethanolic extract in modulating oxidative stress and inflammation, highlighting the dose-dependent nature of GSS ethanolic extract effects. These results align with previous research that has underscored the connection between the response of these biomarkers and the anti-inflammatory and antioxidant properties. In the presence of anti-inflammatory and antioxidants, there is a reduction in the concentrations of TNF-*α*[66], IL-6 [67], and ROS [68], along with decreased expression of iNOS [69], increase in the expression of IκB*α*[70], and rise in SOD concentration [71].

Macroscopic findings and histopathological evaluation of the gastric tissues revealed that the PYL-LIG positive group showed extensive gastric mucosal damage characterized by hemorrhagic lesions, mucosal erosion, and severe inflammation. This outcome aligns with the previously reported studies for induction of gastritis, reflecting the severity of inflammation and mucosal disruption typically observed in such conditions [72].

In our study, the oral administration of GSS ethanolic extract (200&400 mg/kg) resulted in improvements in the gastric tissues. The treated groups with GSS ethanolic extracts in two doses (200&400 mg/kg) exhibited reduced mucosal injury showing only superficial disruptions rather than extensive damage with a reduction in mucosal erosion, focal necrosis, and inflammatory cells in the macroscopic and histopathological evaluations. These results suggest the gastroprotective activity of GSS ethanolic extract in gastritis which supports the reported studies for gastroprotective treatments in gastritis [73].

Molecular docking analysis was performed to identify and compare the interactions between the major metabolites of GSS ethanolic extract with the target proteins. The compounds that gave the best docking score based on the binding free energy and H-bonding with its distance between the amino acid in the receptor and root mean square deviation (RMSD) from the native ligand were soyasaponin I followed by kaempferol-3-*O*-robinoside-7-*O*-rhamnoside. For soyasaponin I, the robust hydrogen bonding and Pi-alkyl interactions with human IL-6, SOD, and human IL-10 indicate its ability to modulate key proteins involved in inflammatory and oxidative stress responses. The interaction with IL-6 suggests it might help regulate inflammatory processes, while its binding to SOD implies potential enhancement of antioxidant defenses. The interactions with IL-10 further support its role in modulating immune responses. Similarly, kaempferol-3-*O*-robinoside-7-*O*-rhamnoside's interactions with the same set of targets highlight its broad-spectrum therapeutic potential. Its ability to form multiple hydrogen bonds and Pi-alkyl interactions with IL-6, SOD, IL-10, HO-1, and Keap-1 suggests it could influence inflammation, oxidative stress, and cellular protection mechanisms. The strong binding energies observed indicate that kaempferol-3-*O*-robinoside-7-*O*-rhamnoside could be an effective modulator of these critical biological pathways, potentially offering protective and regulatory benefits in various disease contexts. These findings support previous studies that highlight the anti-inflammatory and antioxidant properties of soyasaponin I [74] and kaempferol-3-*O*-robinoside-7-*O*-rhamnoside [63], suggesting their potential therapeutic applications in gastritis treatment.

Finally, *Gliricidia sepium* (Jacq.) Kunth. ex. Walp. stem ethanolic extract holds significant promise as a potential treatment for gastritis, primarily due to the presence of its major compounds identified through UHPLC-QTOF-MS/MS analysis, which are recognized for their antioxidative and anti-inflammatory characteristics. Our findings, including the assessment of biomarkers, macroscopic findings, histopathological examination, immunological, and molecular docking results strongly suggest that GSS ethanolic extract can effectively manage gastritis.

## 5 Conclusion

In conclusion, *Gliricidia sepium* (Jacq.) Kunth. ex. Walp. stem ethanolic extract demonstrates significant potential as a viable treatment option for gastritis, driven by the presence of its major compounds with well-established antioxidative and anti-inflammatory properties. This study's comprehensive assessment, ranging from the identification of bioactive constituents to various experimental evaluations, collectively supports the efficacy of GSS ethanolic extract in addressing gastritis-related concerns.

## Consent for publication

All authors of this review have consented for publication.

## Funding

This research did not receive any specific grant from funding agencies in the public, commercial, or not-for-profit sectors.

## CRediT authorship contribution statement

**Mohamed Osama G.:** Software, Methodology, Data curation. **Wafaey Aya A.:** Writing – review & editing, Writing – original draft, Software, Resources, Methodology, Investigation, Formal analysis, Data curation. **El-Hawary Seham S.:** Visualization, Validation, Supervision, Conceptualization. **Kirollos Farid N.:** Writing – review & editing, Visualization, Validation. **El-Rashedy Ahmed A.:** Software, Methodology, Formal analysis, Data curation. **Abdelhameed Mohamed F.:** Writing – review & editing, Visualization, Validation, Software, Resources, Methodology, Investigation, Formal analysis, Data curation. **Abdelrahman Sahar S.:** Software, Methodology, Formal analysis, Data curation. **Ali Alaa M.:** Software, Methodology, Formal analysis, Data curation.

## Declaration of Competing Interest

The authors declare that they have no known competing financial interests or personal relationships that could have appeared to influence the work reported in this paper.

## Data Availability

Data will be made available on request.
