# Supplementary material for: UHPLC-QTOF-MS/MS profiling, molecular networking, and molecular docking analysis of Gliricidia sepium (Jacq.) Kunth. ex. Walp. stem ethanolic extract and its gastroprotective effect on gastritis in rats
Source: Toxicol Rep. 2025 Feb 4;14:101944. doi: 10.1016/j.toxrep.2025.101944 (PMC11848478; doi:10.1016/j.toxrep.2025.101944)
Supplement: Supplementary file 1 — Supplementary material [file mmc1.docx]

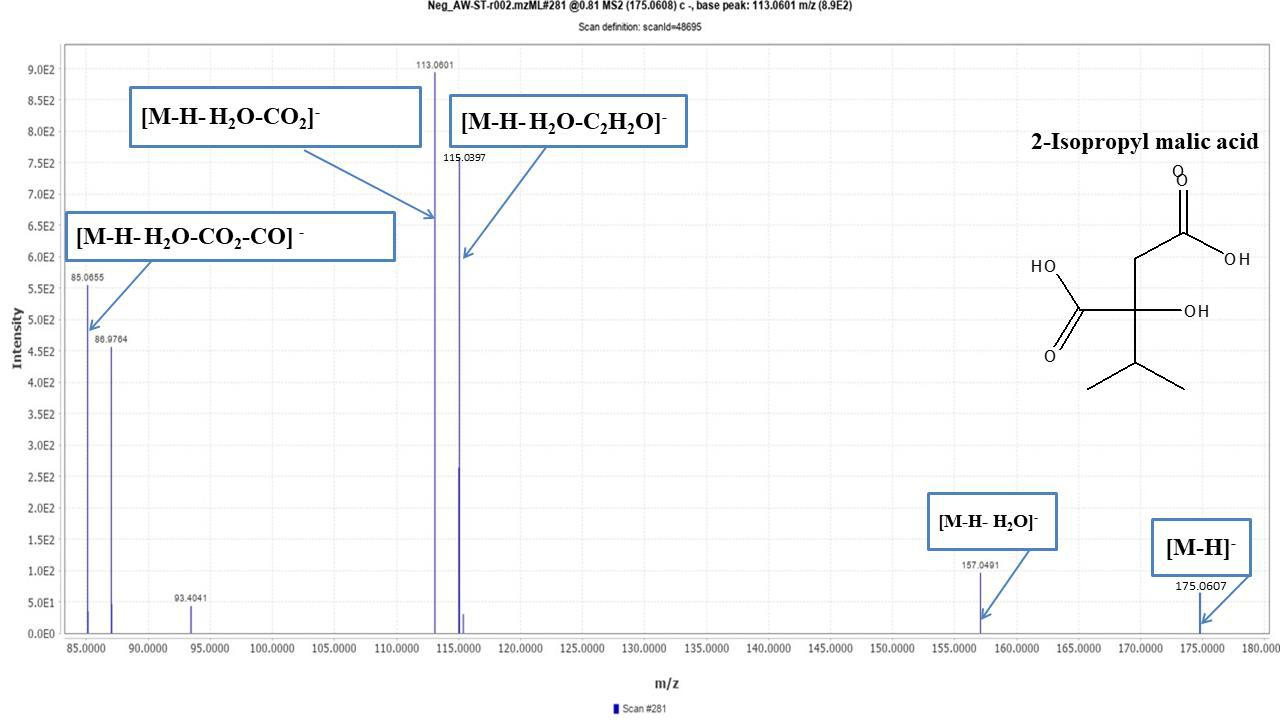


**Fig. S1.** MS/MS spectrum of 2-isopropyl malic acid in *Gliricidia sepium* (Jacq.) Kunth. ex. Walp. stem ethanolic extract.


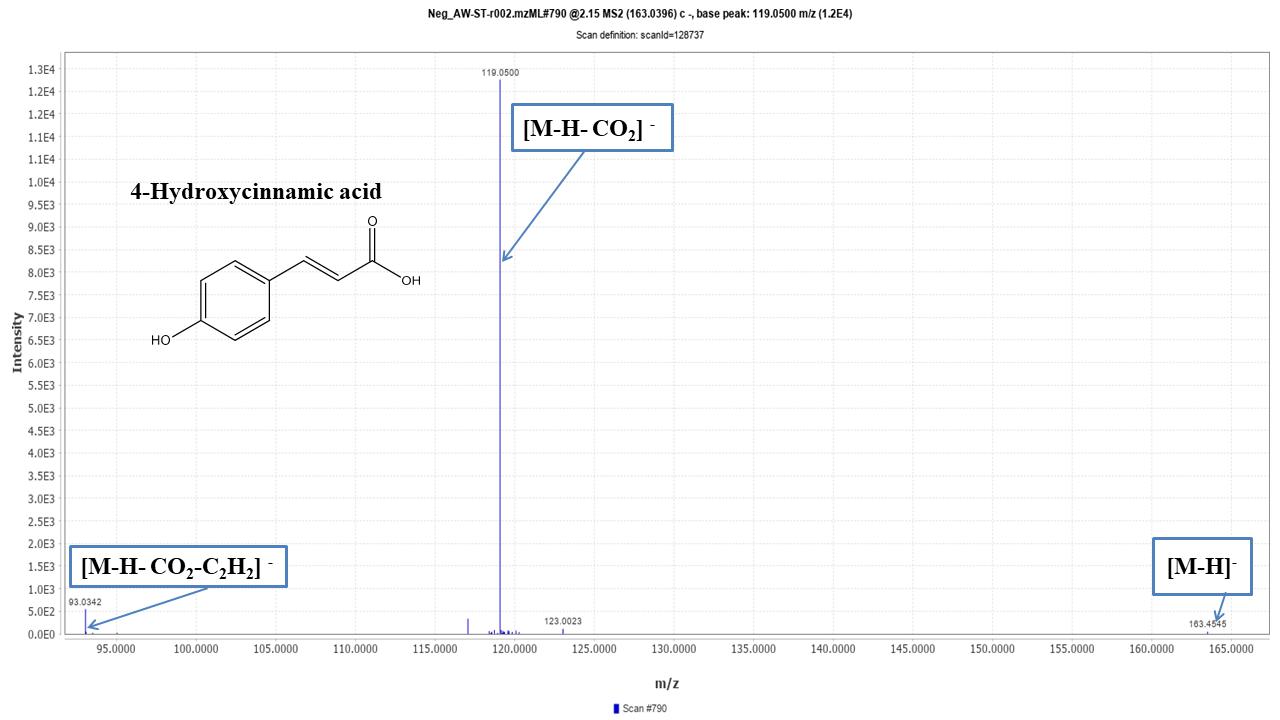


**Fig. S2.** MS/MS spectrum of 4-hydroxycinnamic acid in *Gliricidia sepium* (Jacq.) Kunth. ex. Walp. stem ethanolic extract.


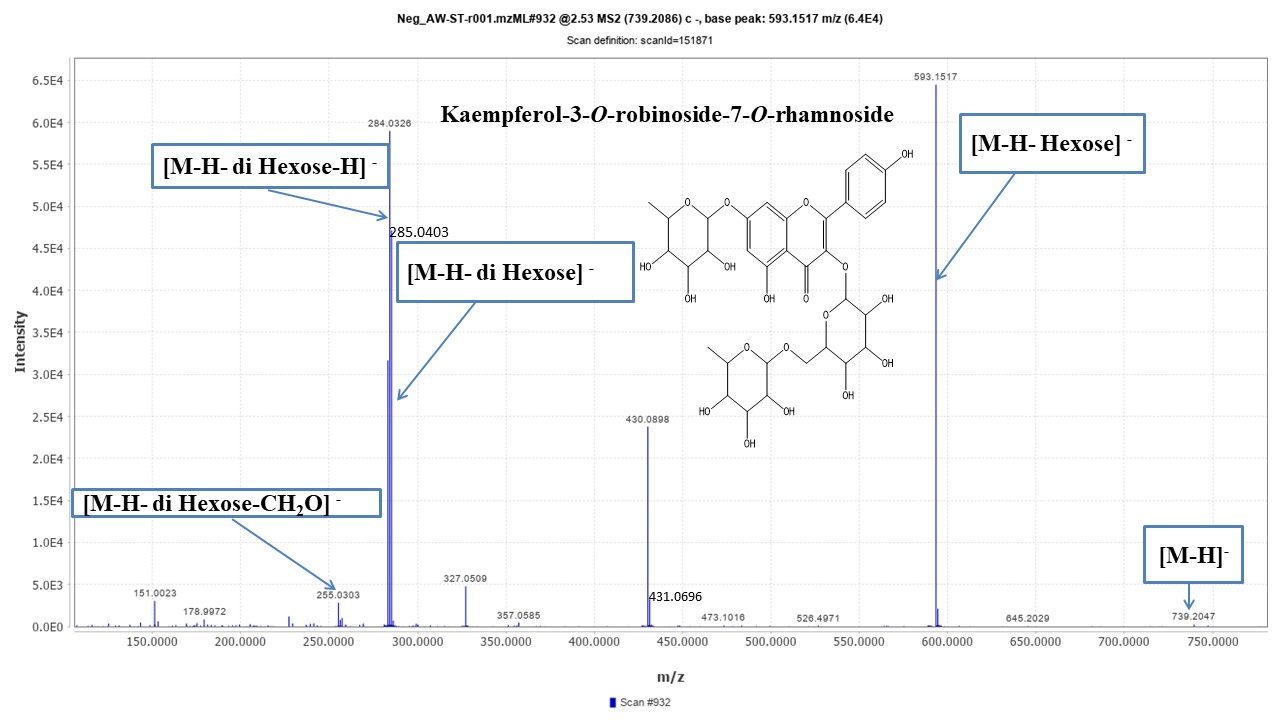


**Fig. S3.** MS/MS spectrum of kaempferol-3-*O*-robinoside-7-*O*-rhamnoside in *Gliricidia sepium* (Jacq.) Kunth. ex. Walp. stem ethanolic extract.


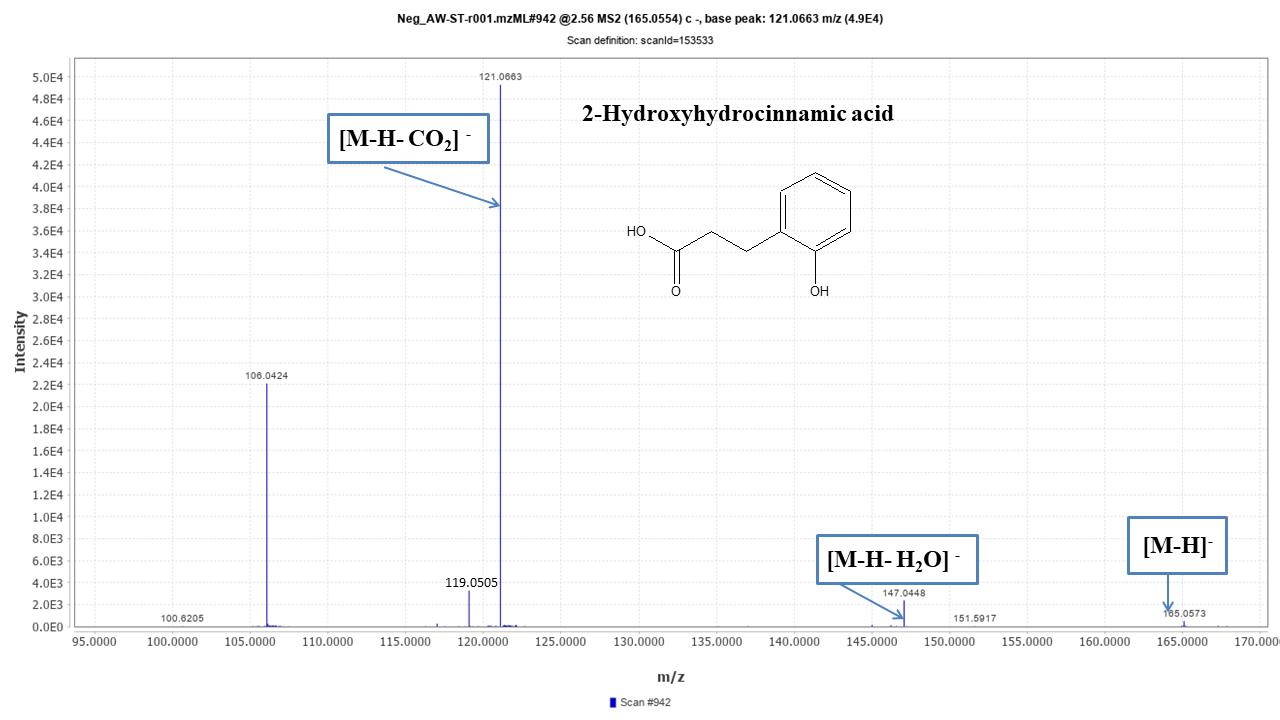


**Fig. S4.** MS/MS spectrum of 2-hydroxyhydrocinnamic acid in *Gliricidia sepium* (Jacq.) Kunth. ex. Walp. stem ethanolic extract.


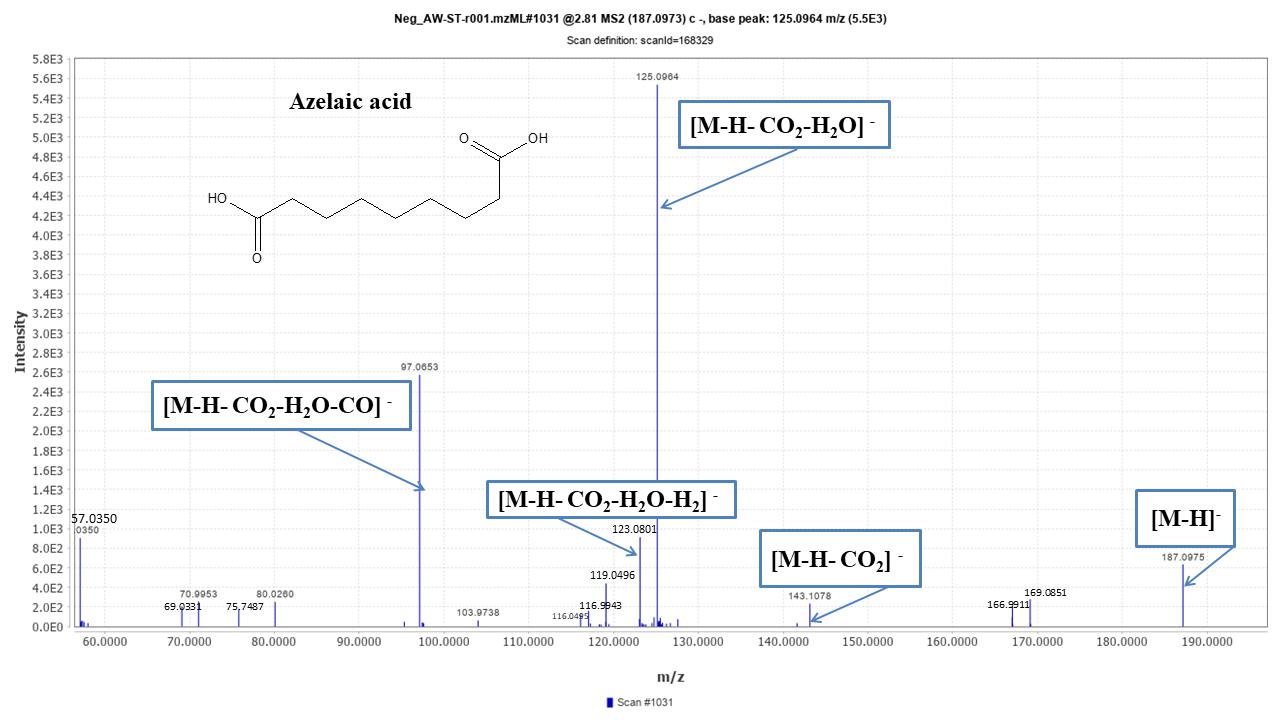


**Fig. S5.** MS/MS spectrum of azelaic acid in *Gliricidia sepium* (Jacq.) Kunth. ex.

Walp. stem ethanolic extract.


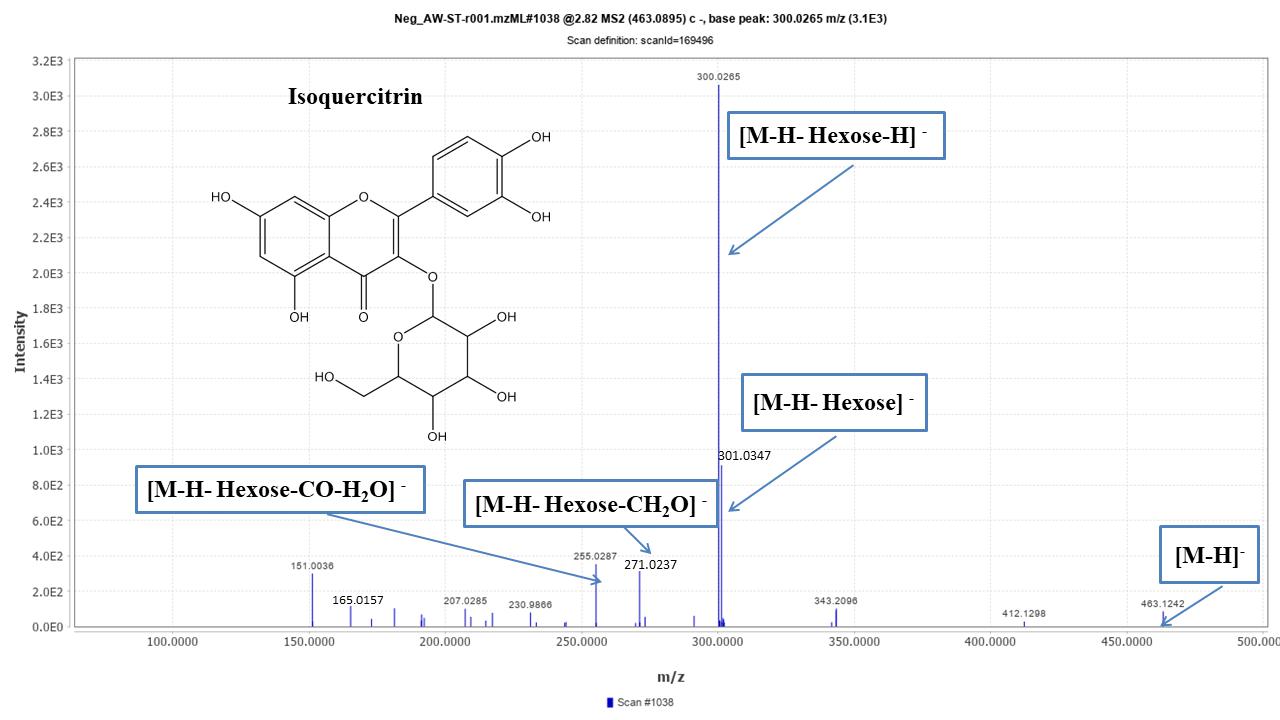


**Fig. S6.** MS/MS spectrum of isoquercitrin in *Gliricidia sepium* (Jacq.) Kunth. ex.

Walp. stem ethanolic extract.


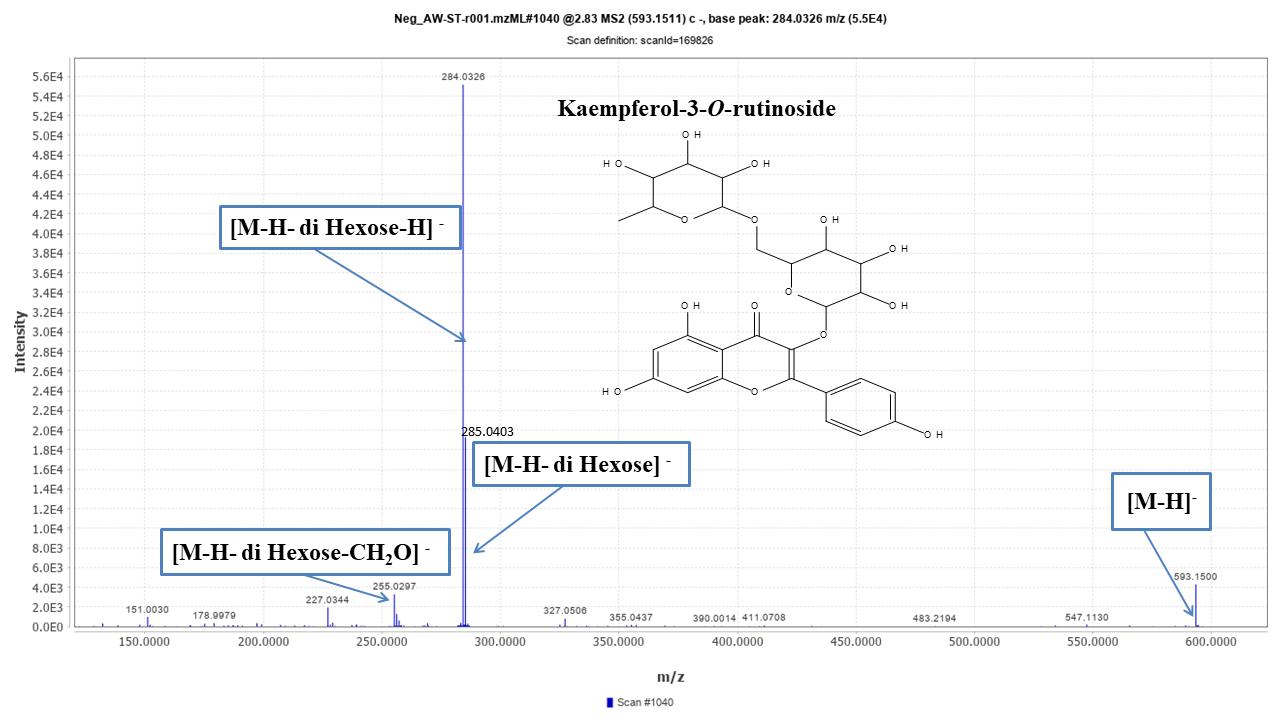


**Fig. S7.** MS/MS spectrum of kaempferol-3-*O*-rutinoside in *Gliricidia sepium* (Jacq.) Kunth. ex. Walp. stem ethanolic extract.


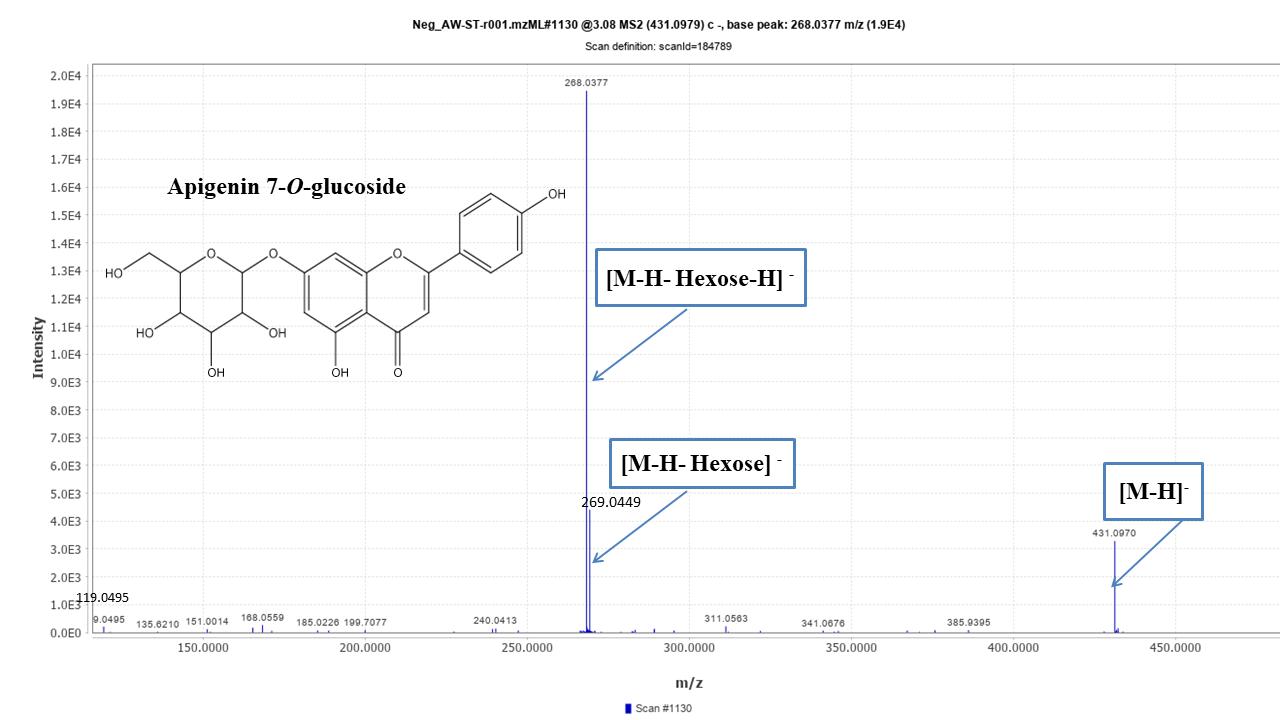


**Fig. S8.** MS/MS spectrum of apigenin 7-*O*-glucoside in *Gliricidia sepium* (Jacq.) Kunth. ex. Walp. stem ethanolic extract.


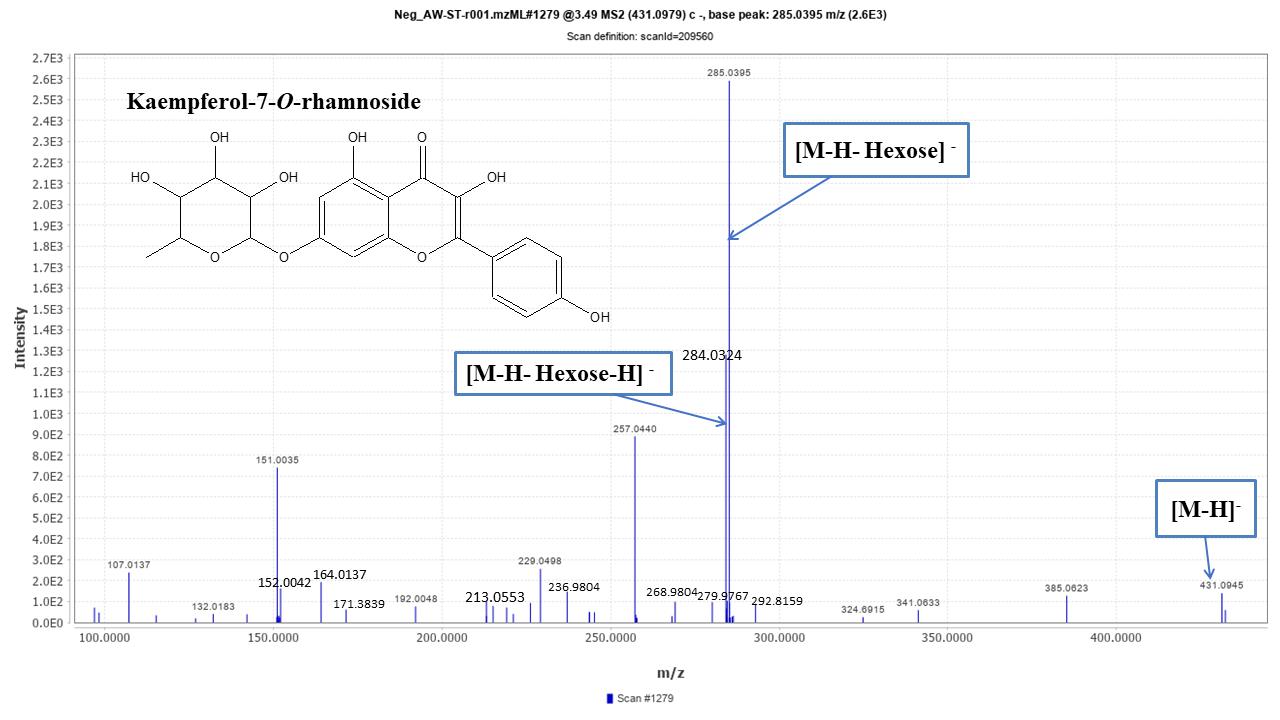


**Fig. S9.** MS/MS spectrum of kaempferol-7-*O*-rhamnoside in *Gliricidia sepium* (Jacq.) Kunth. ex. Walp. stem ethanolic extract.


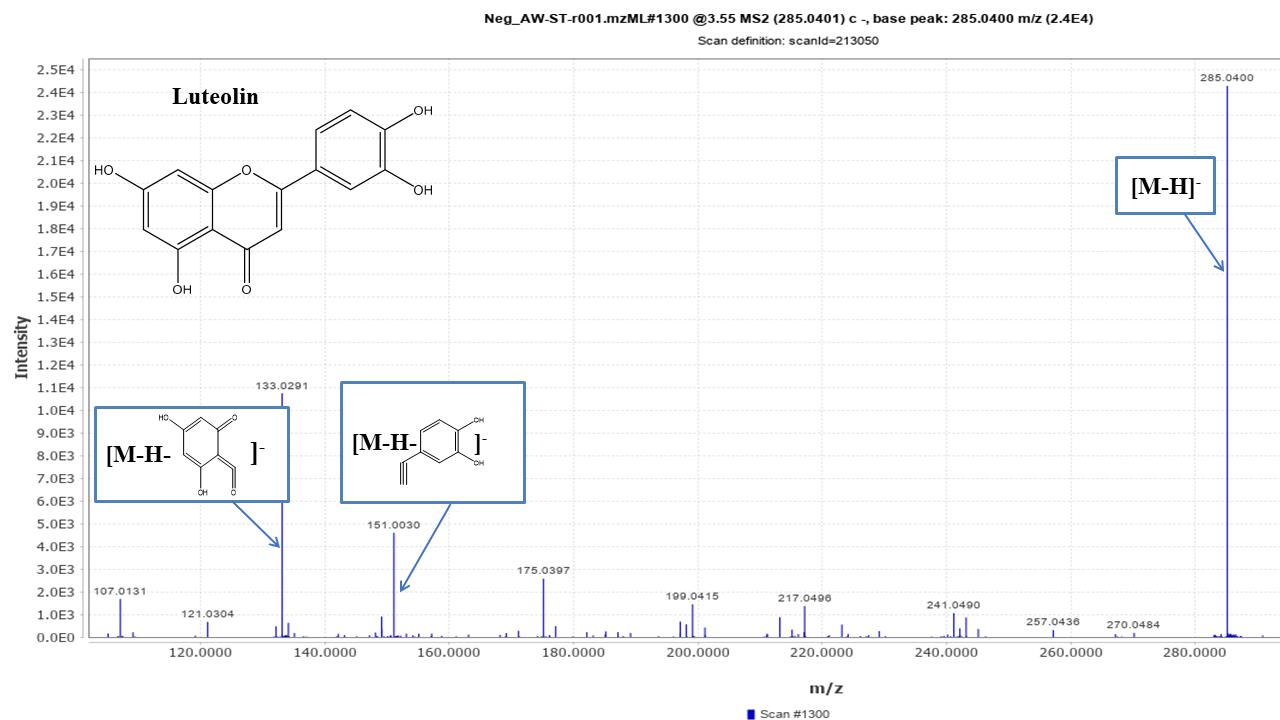


**Fig. S10.** MS/MS spectrum of luteolin in *Gliricidia sepium* (Jacq.) Kunth. ex.

Walp. stem ethanolic extract.


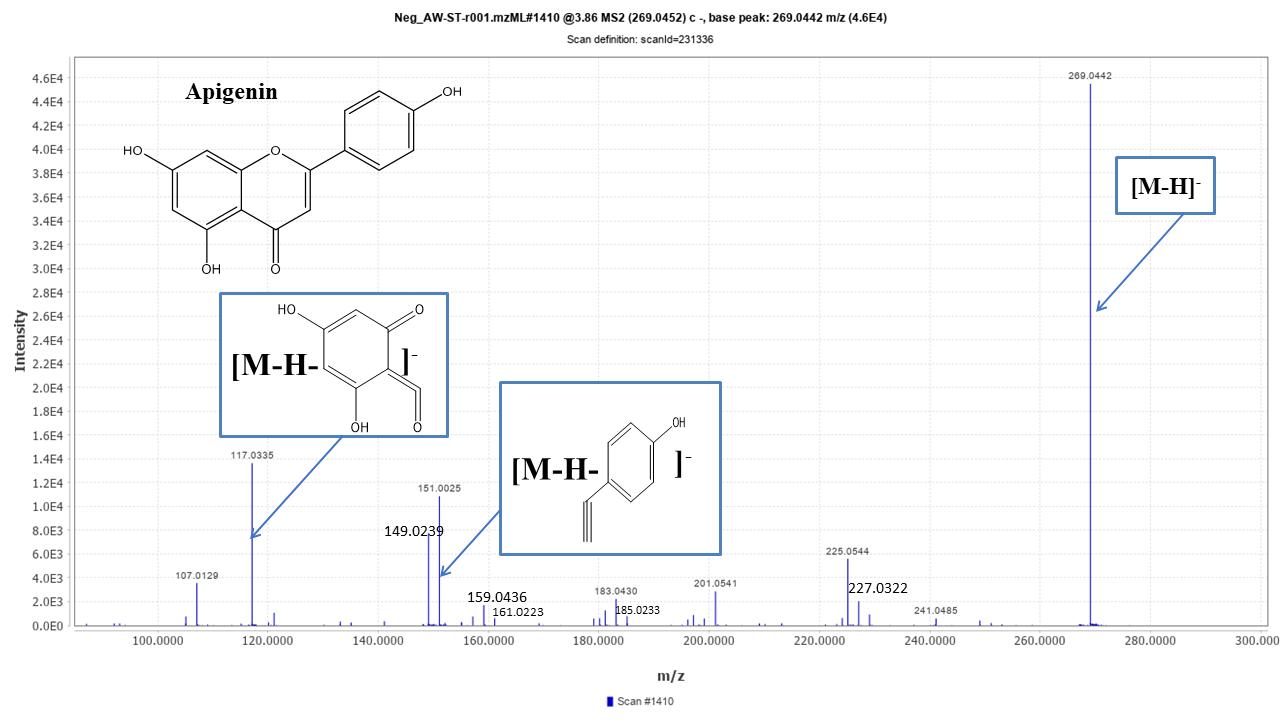


**Fig. S11.** MS/MS spectrum of apigenin in *Gliricidia sepium* (Jacq.) Kunth. ex.

Walp. stem ethanolic extract.


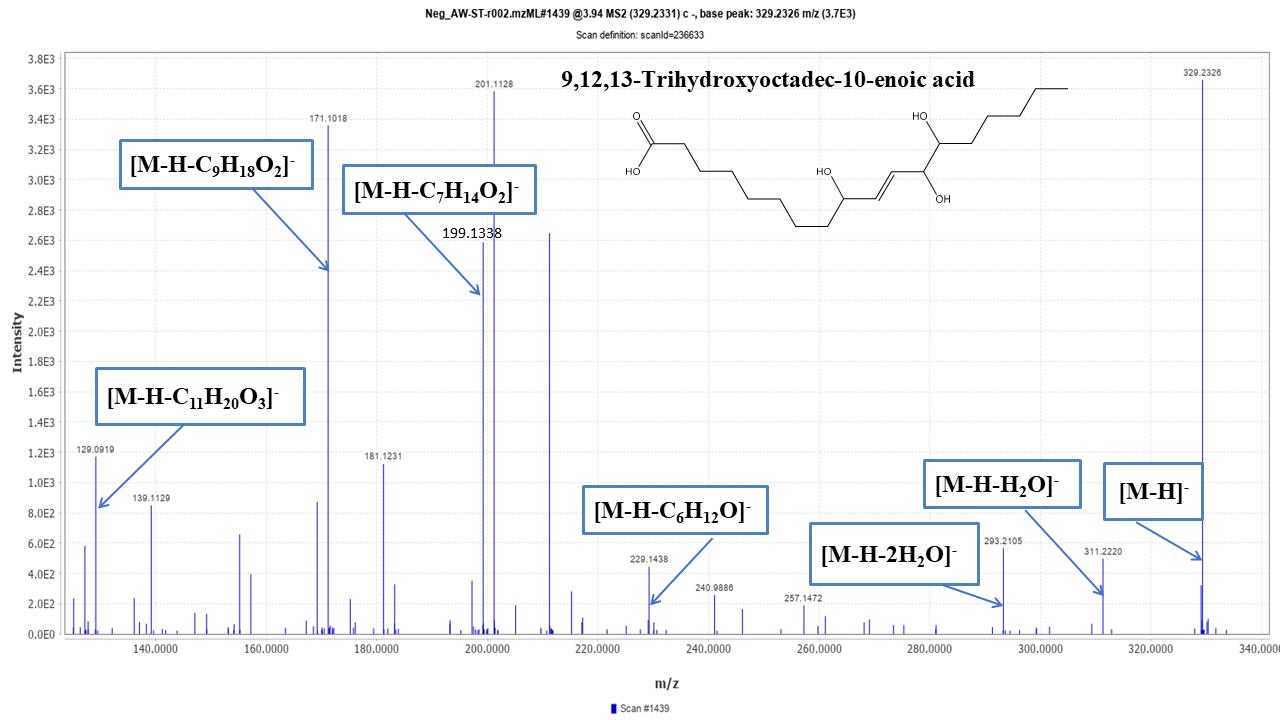


**Fig. S12.** MS/MS spectrum of 9,12,13-trihydroxyoctadec-10-enoic acid in *Gliricidia sepium* (Jacq.) Kunth. ex. Walp. stem ethanolic extract.


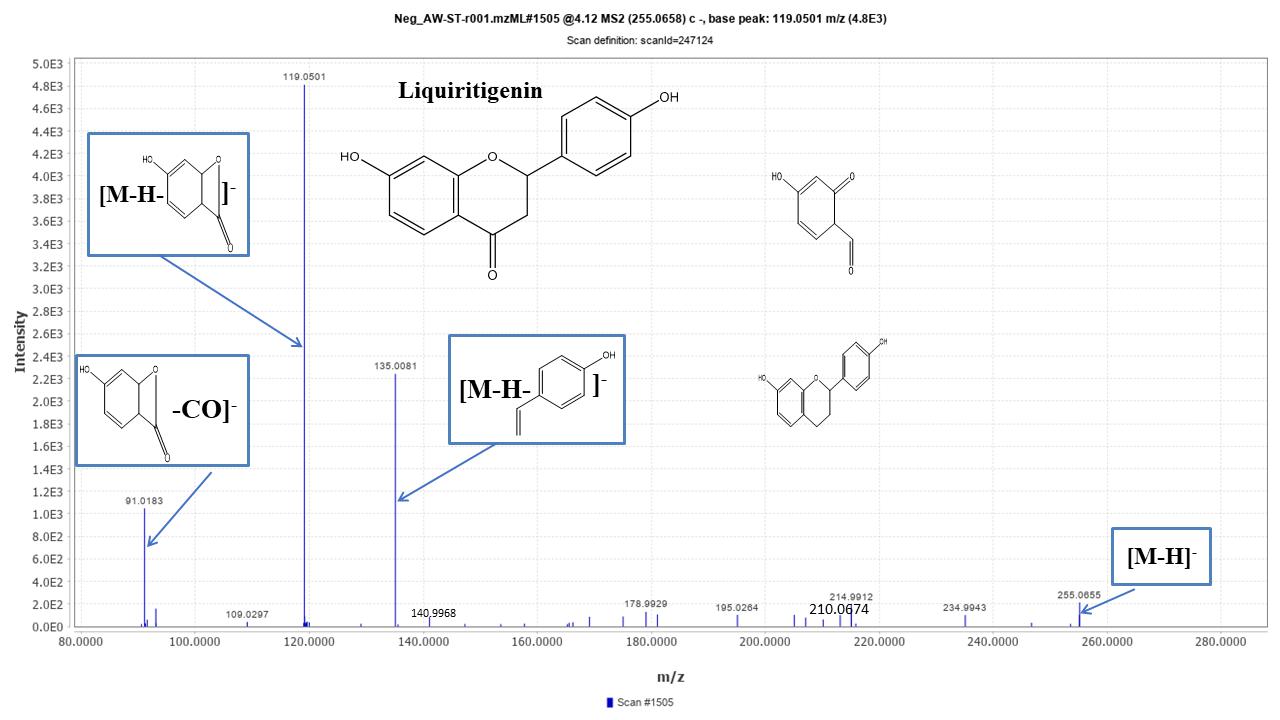


**Fig. S13.** MS/MS spectrum of liquiritigenin in *Gliricidia sepium* (Jacq.) Kunth. ex.

Walp. stem ethanolic extract.


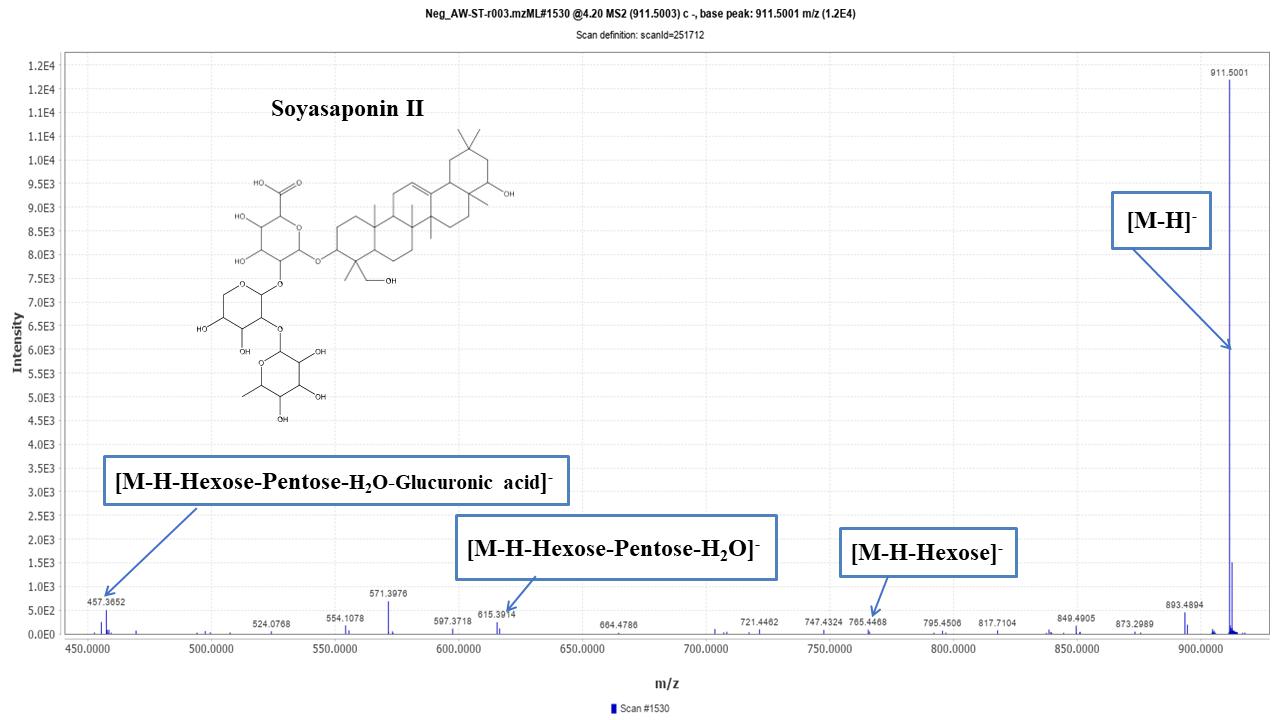


**Fig. S14.** MS/MS spectrum of soyasaponin II in *Gliricidia sepium* (Jacq.) Kunth.

ex. Walp. stem ethanolic extract.


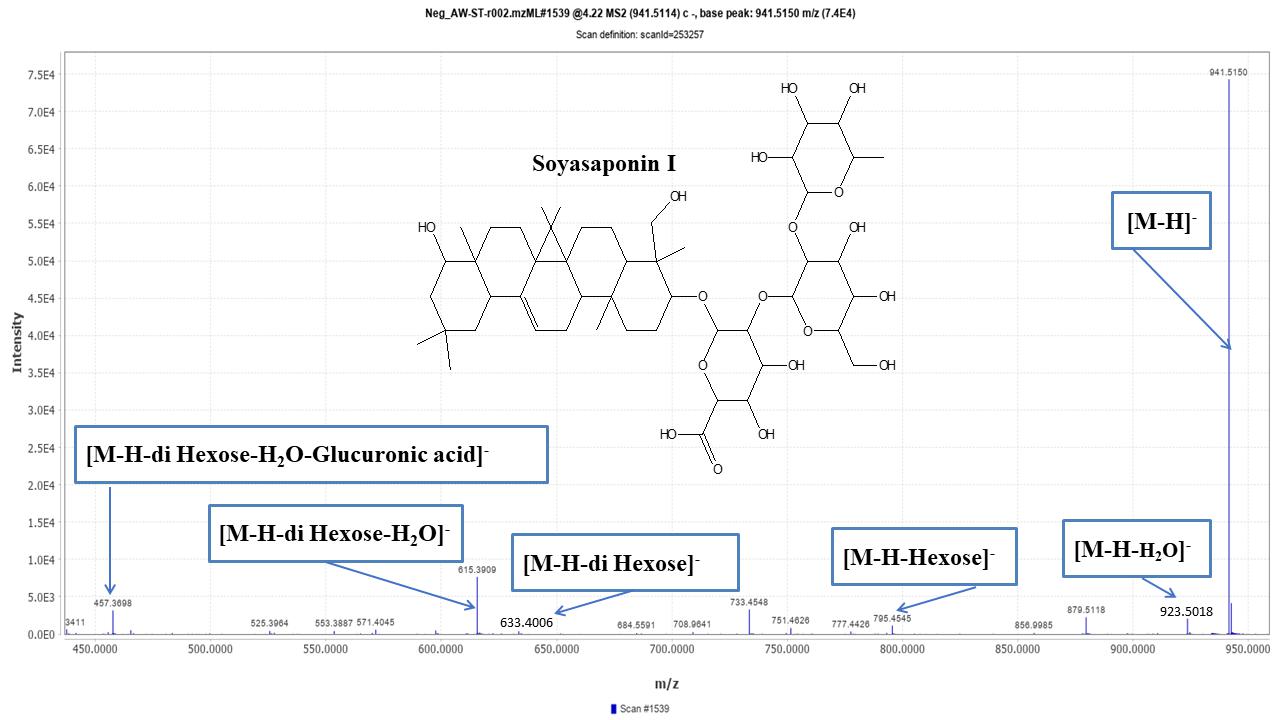


**Fig. S15.** MS/MS spectrum of soyasaponin I in *Gliricidia sepium* (Jacq.) Kunth.

ex. Walp. stem ethanolic extract.


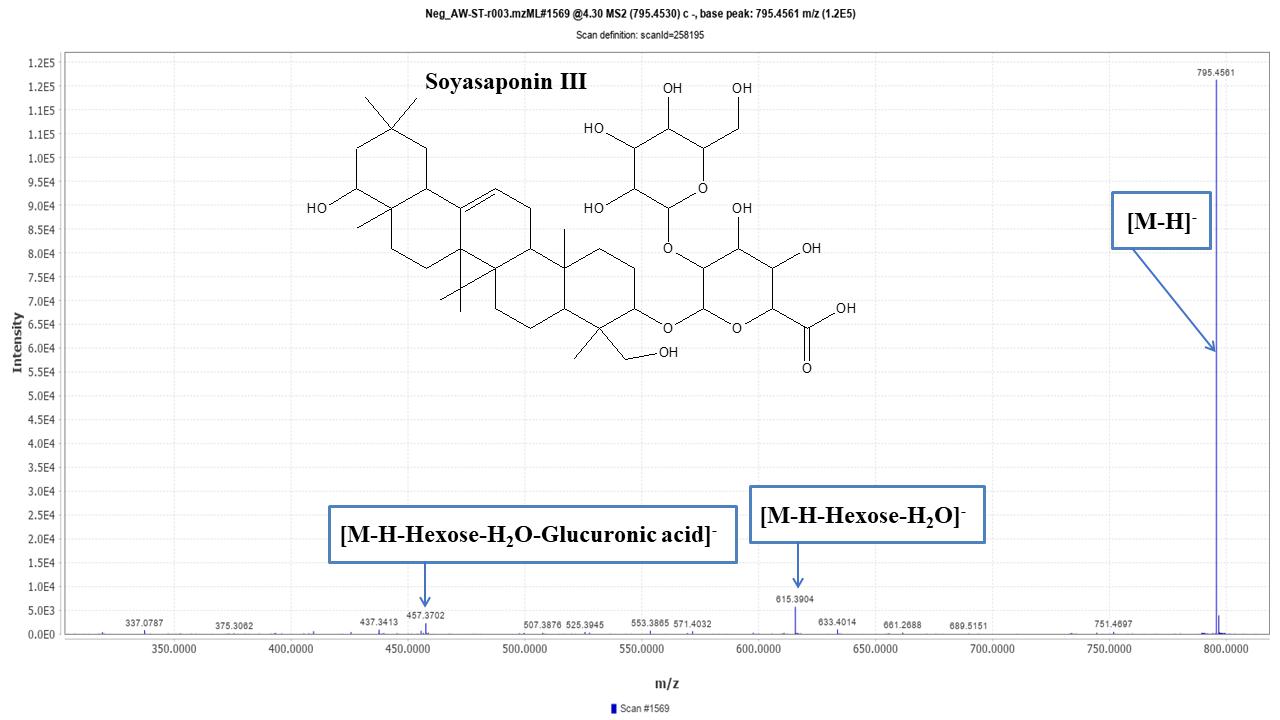


**Fig. S16.** MS/MS spectrum of soyasaponin III in *Gliricidia sepium* (Jacq.) Kunth.

ex. Walp. stem ethanolic extract.


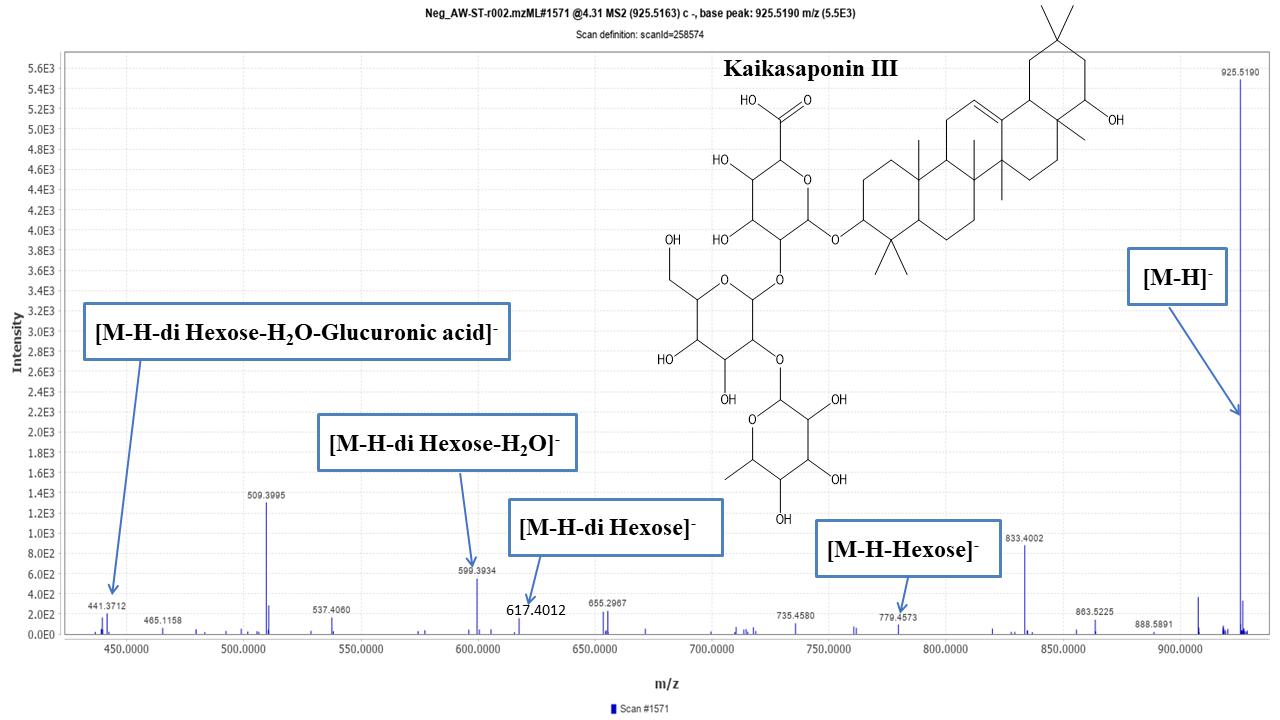


**Fig. S17.** MS/MS spectrum of kaikasaponin III in *Gliricidia sepium* (Jacq.) Kunth.

ex. Walp. stem ethanolic extract.


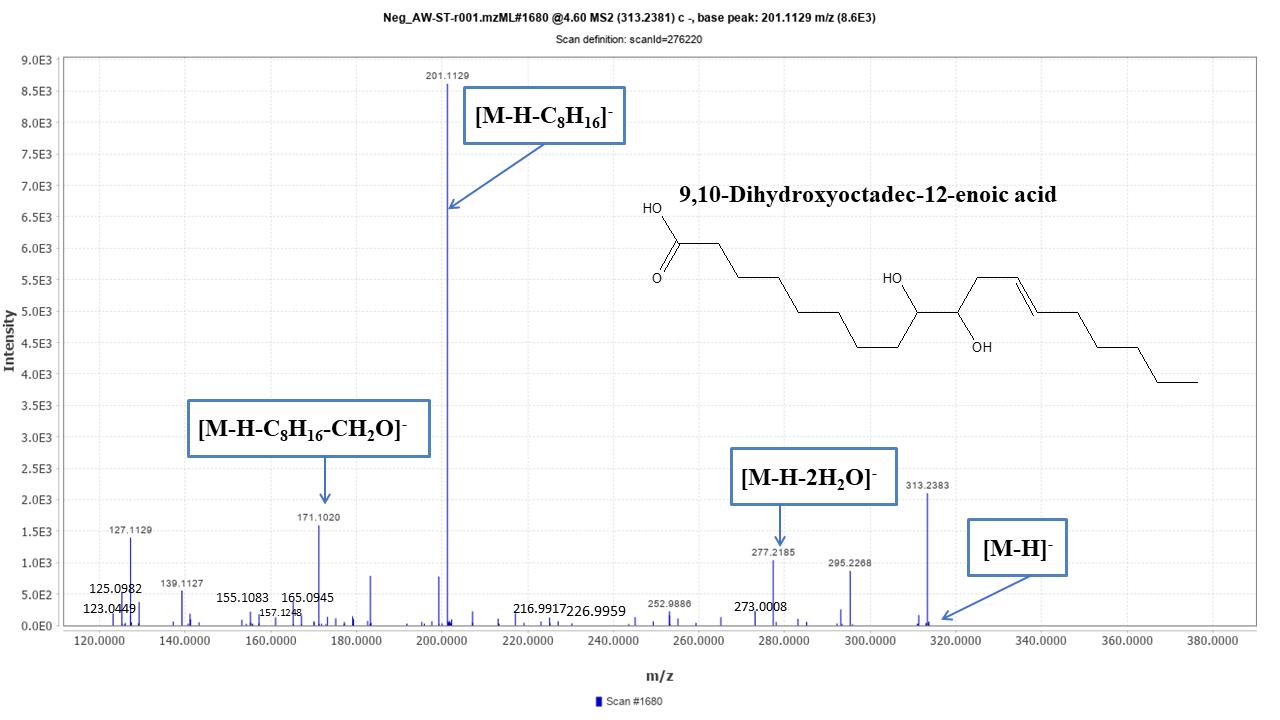


**Fig. S18.** MS/MS spectrum of 9,10-dihydroxyoctadec-12-enoic acid in *Gliricidia* *sepium* (Jacq.) Kunth. ex. Walp. stem ethanolic extract.


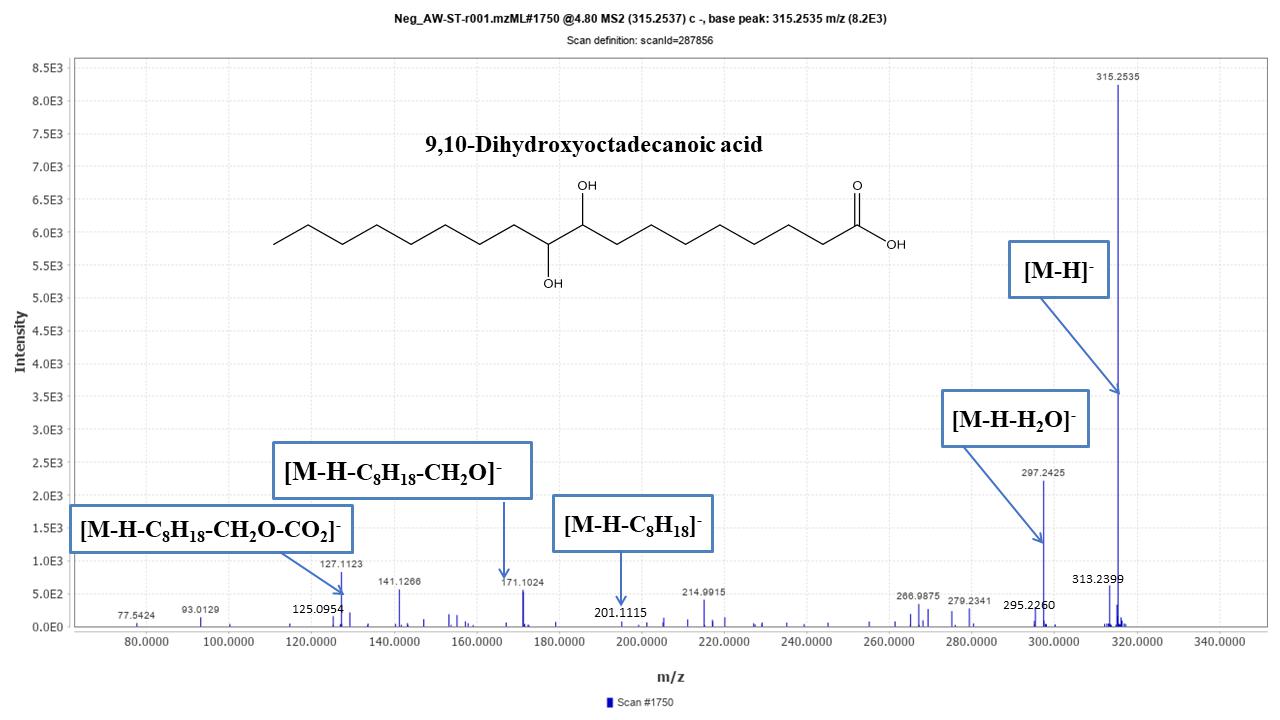


**Fig. S19.** MS/MS spectrum of 9,10-dihydroxyoctadecanoic acid in *Gliricidia* *sepium* (Jacq.) Kunth. ex. Walp. stem ethanolic extract.


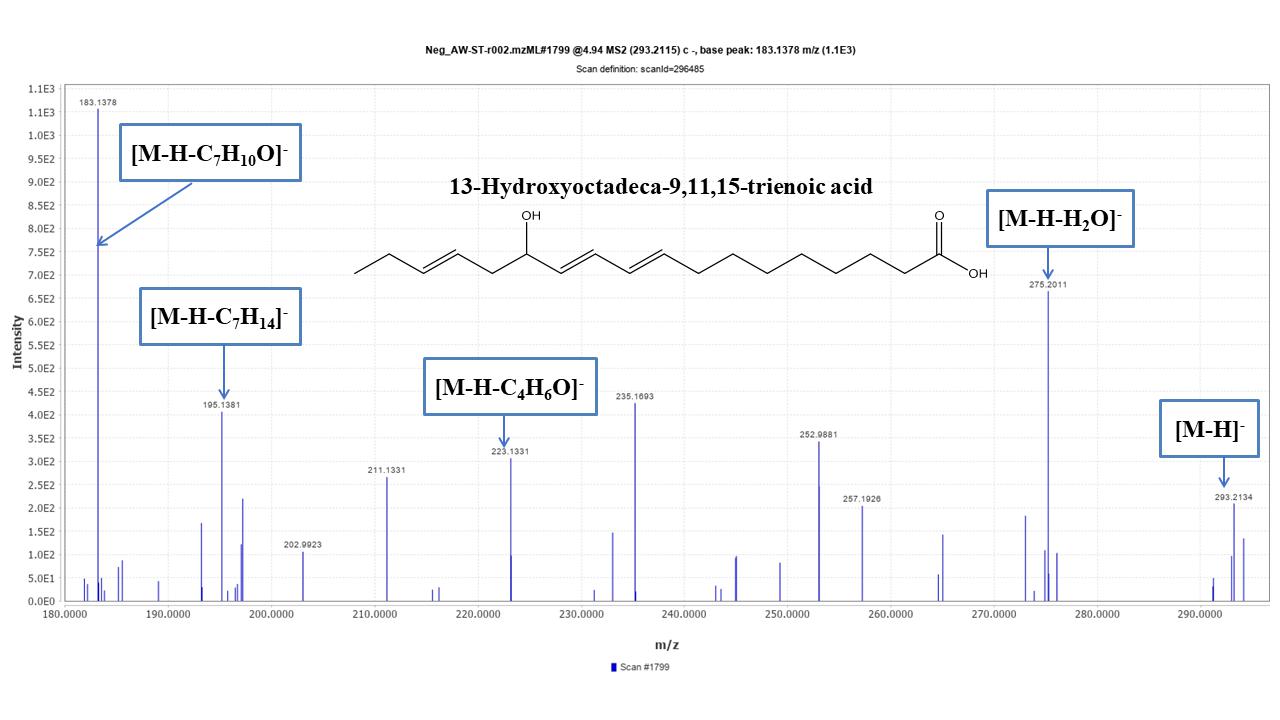


**Fig. S20.** MS/MS spectrum of 13-hydroxyoctadeca-9,11,15-trienoic acid in *Gliricidia sepium* (Jacq.) Kunth. ex. Walp. stem ethanolic extract .


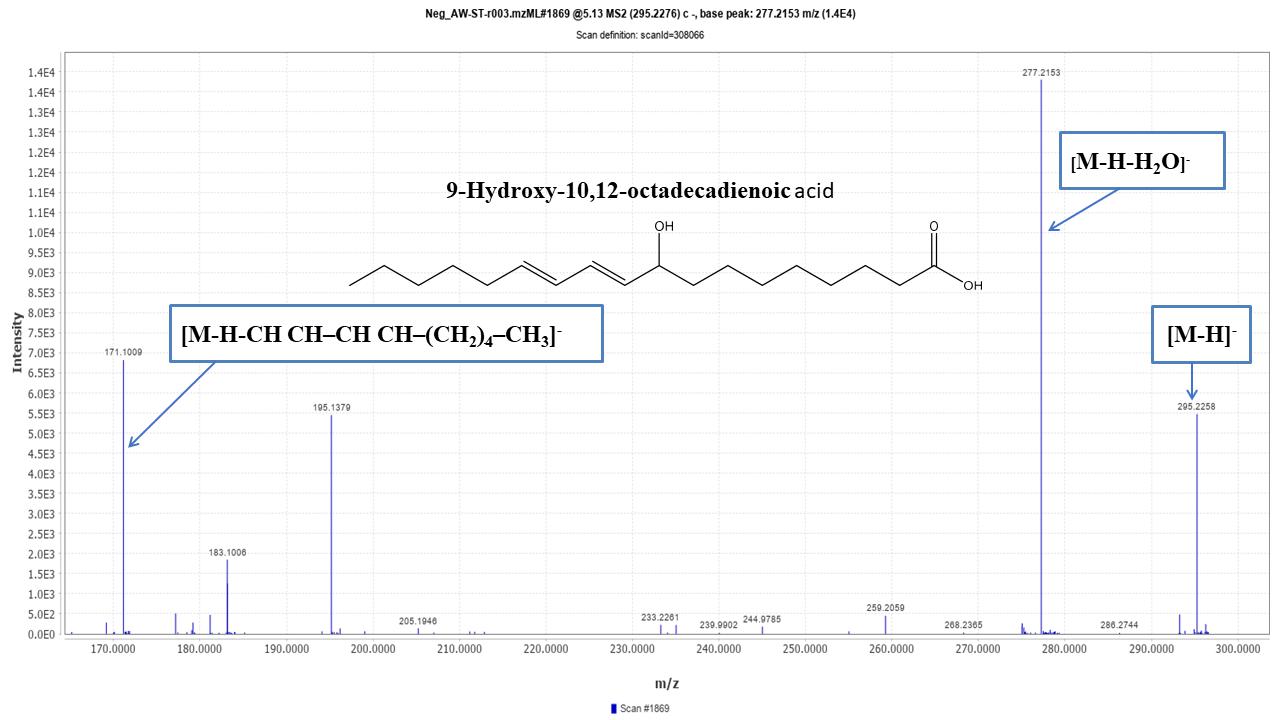


**Fig. S21.** MS/MS spectrum of 9-hydroxy-10,12-octadecadienoic acid in *Gliricidia* *sepium* (Jacq.) Kunth. ex. Walp. stem ethanolic extract.


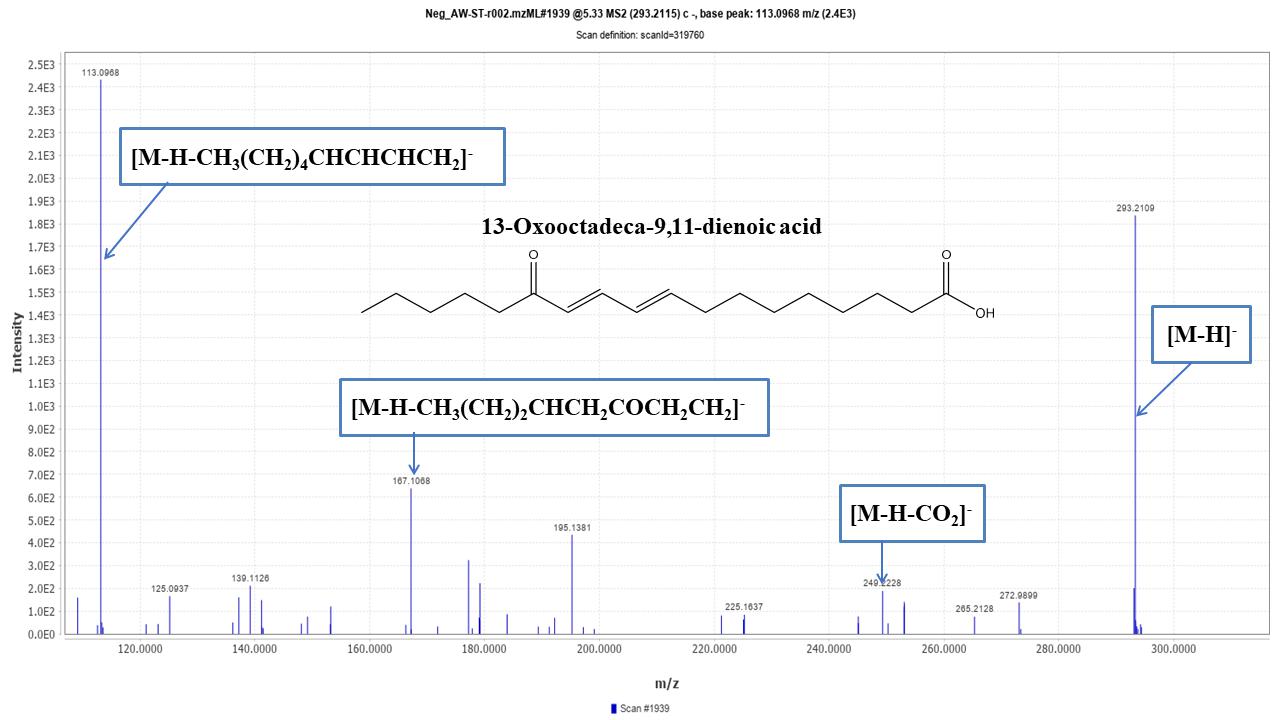


**Fig. S22.** MS/MS spectrum of 13-oxooctadeca-9,11-dienoic acid in *Gliricidia* *sepium* (Jacq.) Kunth. ex. Walp. stem ethanolic extract.


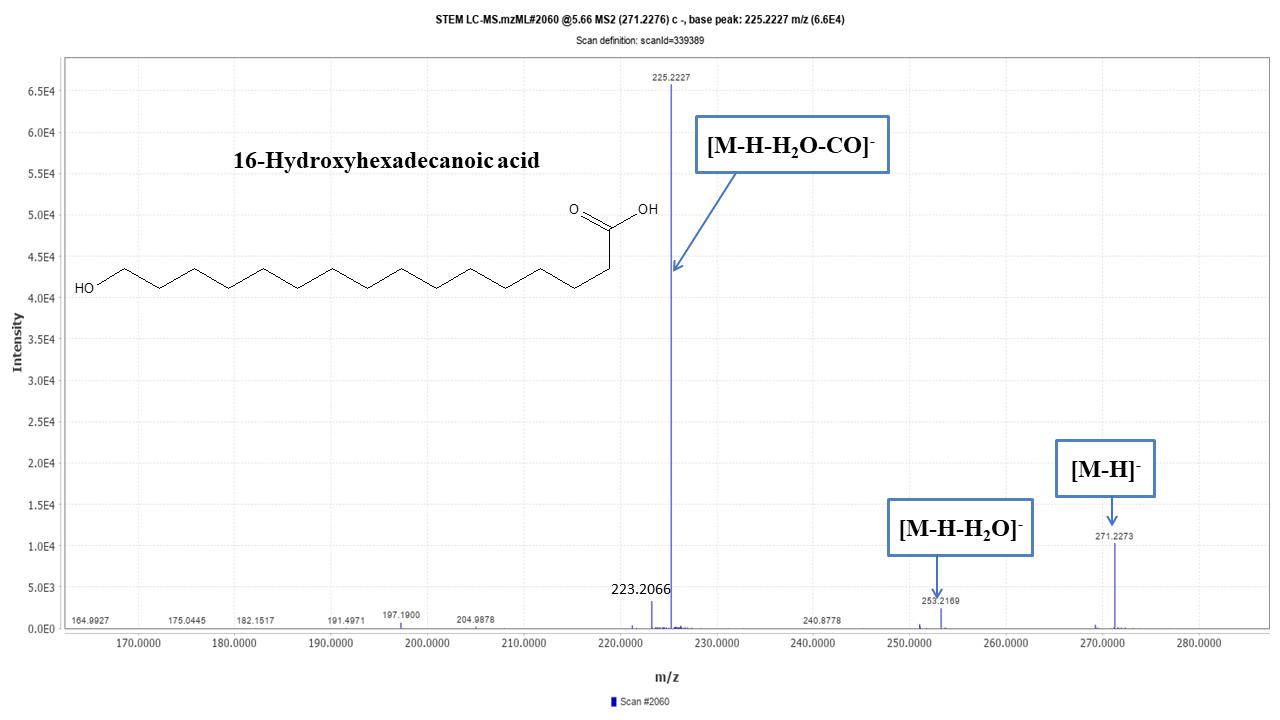


**Fig. S23.** MS/MS spectrum of 16-hydroxyhexadecanoic acid in *Gliricidia sepium* (Jacq.) Kunth. ex. Walp. stem ethanolic extract.
